# Gene essentiality landscape and druggable oncogenic dependencies in herpesviral primary effusion lymphoma

**DOI:** 10.1038/s41467-018-05506-9

**Published:** 2018-08-15

**Authors:** Mark Manzano, Ajinkya Patil, Alexander Waldrop, Sandeep S. Dave, Amir Behdad, Eva Gottwein

**Affiliations:** 1 Department of Microbiology-Immunology, Feinberg School of Medicine, Northwestern University, Chicago, IL 60611 USA; 2 Duke Cancer Institute and Center for Genomic and Computational Biology, Duke University, Durham, NC 27708 USA; 3 Department of Pathology, Feinberg School of Medicine, Northwestern University, Chicago, IL 60611 USA

## Abstract

Primary effusion lymphoma (PEL) is caused by Kaposi’s sarcoma-associated herpesvirus. Our understanding of PEL is poor and therefore treatment strategies are lacking. To address this need, we conducted genome-wide CRISPR/Cas9 knockout screens in eight PEL cell lines. Integration with data from unrelated cancers identifies 210 genes as PEL-specific oncogenic dependencies. Genetic requirements of PEL cell lines are largely independent of Epstein-Barr virus co-infection. Genes of the NF-κB pathway are individually non-essential. Instead, we demonstrate requirements for IRF4 and MDM2. PEL cell lines depend on cellular cyclin D2 and c-FLIP despite expression of viral homologs. Moreover, PEL cell lines are addicted to high levels of MCL1 expression, which are also evident in PEL tumors. Strong dependencies on cyclin D2 and MCL1 render PEL cell lines highly sensitive to palbociclib and S63845. In summary, this work comprehensively identifies genetic dependencies in PEL cell lines and identifies novel strategies for therapeutic intervention.

## Abstract (web summary)

Our understanding of the genetics and treatment options for primary effusion lymphoma (PEL) is lacking. Here the authors performed in vitro CRISPR/Cas9 knock-out screens to identify 210 PEL-specific oncogenic genes and report strategies for therapeutic intervention.

## Introduction

The human oncogenic γ-herpesvirus Kaposi’s sarcoma-associated herpesvirus (KSHV) causes primary effusion lymphoma (PEL), Kaposi’s sarcoma, and a subtype of the lymphoproliferative disorder multicentric Castleman’s disease^1–4^. PELs typically occur in the context of immunosuppression and present as clonal effusions of post-germinal center B cells into body cavities^5^. The current treatment regimen for PEL is standard chemotherapy and, in HIV/AIDS-associated cases, combination antiretroviral therapy^6^. Despite this, prognosis of this disease remains poor, with a median survival time of 6 months^7^. Thus, better treatment alternatives are urgently needed.

Genetic loci that are translocated or mutated in other B cell lymphomas, such as the proto-oncogene MYC or tumor suppressor protein p53 (TP53), are typically unaltered in PEL^8–10^. Instead, the defining feature of this cancer is the presence of KSHV in each tumor cell. In the vast majority of cells, KSHV undergoes latency, with expression of only a small number of viral proteins, including latent nuclear antigen (LANA), a viral interferon regulatory factor (vIRF3/LANA2), viral homologs of D-type cyclins (vCYC) and FLICE inhibitory protein/c-FLIP/CFLAR (vFLIP), and a cluster of viral microRNAs. Most PEL tumors (~80%) are co-infected with the oncogenic γ-herpesvirus Epstein-Barr virus (EBV), pointing to a role of EBV in PEL^5^. A role for EBV is experimentally supported by the finding that introduction of EBV into EBV-negative PEL cell lines increases xenograft formation in severe combined immune deficiency mice^11^. KSHV also enhances EBV-associated B cell lymphomagenesis in a humanized mouse model^12^. Nevertheless, KSHV is clearly the main oncogenic driver of PEL because EBV-negative cases exist and PEL-derived cell lines require the constitutive expression of at least LANA, vFLIP, and vIRF3, regardless of EBV co-infection^13–15^. Whether EBV contributes to the survival and proliferation of dually KSHV- and EBV-infected PEL cell lines is unknown.

The current model of PEL oncogenesis suggests critical roles for inhibition of the p53 family of tumor suppressors and the constitutive activation of nuclear factor kappa B (NF-κB), cytokine, and PI3K/Akt/mTOR signaling pathways. The viral LANA protein is critical, as it mediates the episomal maintenance of the KSHV genome during cell division. LANA also forms a complex with p53 and the p53 ubiquitin ligase MDM2, and thereby blocks p53 function^16^. The function of p53, and the related p73, can be reactivated in PEL cells with Nutlin-3a, which disrupts the p53/MDM2 and p53/MDM2/LANA complexes and triggers apoptosis and cell cycle arrest^9,16–18^. In addition to LANA, vIRF3 also binds and inhibits p53^19^. The essentiality of vFLIP in PEL cell lines is thought to be due to its direct interaction with the NEMO (encoded by *IKBKG*) subunit of the inhibitor of κB kinase (IKK) complex, resulting in the constitutive activation of pro-survival NF-κB signaling^20–24^. PEL cell lines have furthermore been reported to depend on autocrine and paracrine signaling by a KSHV homolog of *IL6* (vIL-6) and cellular cytokines, which activate Jak/Stat signaling^25^. PEL cell lines are sensitive to inhibitors of PI3K and mTOR and thus addicted to high levels of PI3K/Akt/mTOR activity^26,27^, although which viral genes are responsible for this phenotype in PEL cells is unknown. The role of vCYC expression during latency in PEL remains unclear. vCYC drives cell cycle progression following ectopic expression, but differs from cellular D-type cyclins by its preference for cyclin-dependent kinase 6 (CDK6) as a binding partner^28^. vCYC/CDK6 complexes furthermore exhibit an extended substrate range and are relatively refractory to inhibition by CDK inhibitors^29^.

Gene expression profiling places the transcriptome of PEL cell lines and tumors closest to that of plasma cell neoplasms, most notably multiple myeloma^30–32^. Accordingly, PELs express high levels of the transcription factor interferon regulatory factor 4 (IRF4), a critical oncogene in multiple myeloma^33^. More recently, PEL cell lines were suggested to require an IKZF1-IRF4-MYC transcriptional axis, which renders them susceptible to the immunomodulatory drugs (IMiDs) lenalidomide and pomalidomide, due to degradation of IKZF1 and a consequent loss of IRF4 expression^34^.

Our current understanding of which host genes are critical in PEL is likely incomplete and based largely on candidate approaches. Recently, CRISPR/Cas9 gene editing has emerged as a powerful platform for unbiased genome-wide loss-of-function screening^35,36^. Here we utilize genome-wide CRISPR screens for the comprehensive identification of single-gene dependencies in PEL-derived cell lines. We integrate resulting data with a newly generated dataset from multiple myeloma and published screens from 15 other cancer cell types. Our analyses define 210 non-housekeeping genes as PEL-specific oncogenic dependencies (PSODs). Our data identify novel single-gene addictions. In-depth validation experiments demonstrate a strong requirement of PEL cells for *IRF4*, *MDM2*, *CCND2*, and *MCL1*, all of which are druggable, and for *CFLAR*. The newly identified requirements of PEL for cyclin D2 and c-FLIP are surprising given that KSHV expresses homologs of these proteins (vCYC and vFLIP). We furthermore show that MCL1 is highly expressed in PEL tumors and that MCL1 inhibition offers an effective therapeutic strategy. In sum, our work achieves a detailed understanding of the genetic requirements of PEL cell lines and provides important leads for new lines of investigation and novel therapeutic strategies.

## Results

### Genome-wide CRISPR/Cas9 knockout screens in PEL cell lines

**Fig. 1:**
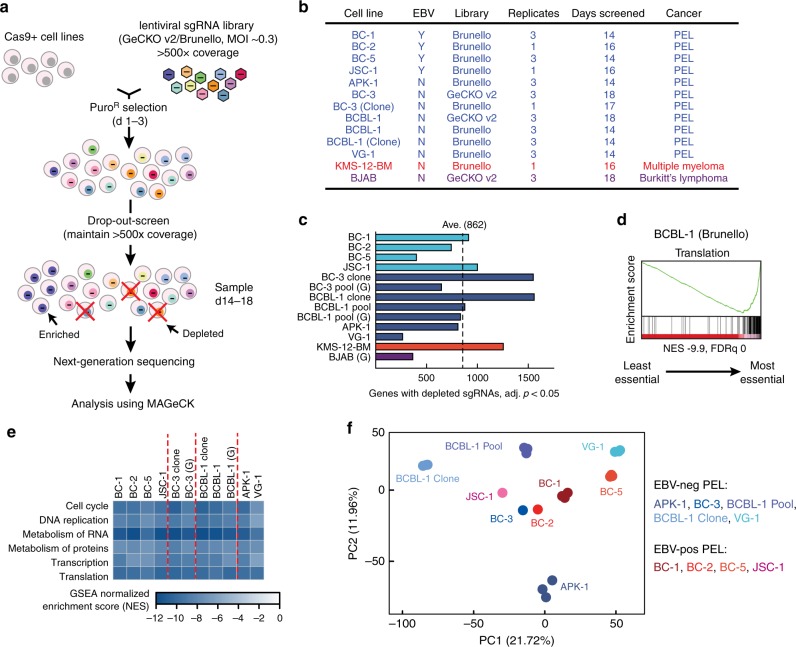
Genome-wide CRISPR knockout screens in PEL cell lines. **a** Experimental outline. Cas9-expressing cell pools or clones were infected with the lentiviral GeCKO v2 or Brunello sgRNA libraries. After complete puromycin selection, cells were split every 2–3 days and maintained at 500× sgRNA coverage. After 14–18 days, sgRNA composition was analyzed by Illumina sequencing and MAGeCK. **b** Cell lines and conditions used in this study. **c** Numbers of genes with significantly depleted sgRNAs in each screen (adj. *p* value < 0.05). “G” indicates cells were screened with GeCKO v2 library; all others were screened with Brunello. Cyan: EBV(+) PEL cells; blue: EBV(−) PEL cells; red: multiple myeloma; purple: Burkitt’s lymphoma. **d** Representative gene set enrichment analysis (GSEA) from BCBL-1 Cas9 clone screened using Brunello library. Genes were ranked by sgRNA depletion scores, with genes with depleted sgRNAs at the right end of the *x*-axis. NES normalized enrichment score, FDRq FDR-adjusted *p* value. **e** Heatmap of GSEA NESs of housekeeping pathways (Reactome) in all PEL cell lines (see Supplementary Data 7). Screens designated “G” used GeCKO v2 library. **f** Principal component analysis of normalized sgRNA reads from EBV(−) (blue shades) or EBV(+) PEL cells (red shades). sgRNAs from genes that have adj. *p* < 0.05 in at least one cell line were examined. Only data from Brunello screens were considered

We performed genome-wide CRISPR/Cas9 knockout screens for essential host genes in eight PEL cell lines, including four that were co-infected with EBV (Fig. 1a, b). As a control for B cell malignancies of non-viral etiology, we similarly screened the commonly used B cell line BJAB and the multiple myeloma cell line KMS-12-BM. Multiple myeloma shares plasma cell differentiation status with PEL^31,32^. Cas9-expressing cell pools and/or cell clones were infected with single guide RNA (sgRNA) libraries^37,38^ and cultured for 2–3 weeks to allow sufficient time for the depletion of cells with inactivated essential and/or fitness genes. sgRNA composition was assessed by Illumina sequencing and compared to input libraries using MAGeCK^39^. The detailed experimental workflow and conditions are summarized in Fig. 1a, b, Supplementary Data 1–6, and Methods. We observed highly significant depletion of numerous sgRNAs but only few enrichments, indicating the existence of many dependencies but few expressed genetic liabilities in cultured PEL cells (Supplementary Figure. 1a, b). Our screens identified on average 862 genes with false discovery rate (FDR)-adjusted (adj.) *p* values of sgRNA depletion (adj. *p*) < 0.05 (Fig. 1c), similar to results from CRISPR screens reported in non-PEL cancer cell lines^40–43^. This cutoff may include both genes that are strictly essential and those that are non-essential but confer increased fitness. We therefore refer to genes that meet this cutoff as “gene dependencies.” Screens performed in cell clones selected for optimal editing identified most gene dependencies (Fig. 1c and below). Therefore, variation in the numbers of identified gene dependencies likely reflects the sensitivity of individual screens due to variable editing efficiencies. Specifically, screens in BJAB, VG-1, and BC-5 performed relatively poorly. Gene set enrichment analyses (GSEAs)^44^ of all datasets revealed the expected striking depletion of sgRNAs for genes with housekeeping functions (Fig. 1d, e, and Supplementary Data 7). In conclusion, our CRISPR knockout screens effectively identified genetic dependencies in PEL cell lines.

### Genetic requirements of EBV-negative and -positive PEL cell lines

The high rate of EBV co-infection in PEL tumors and increased tumor formation of KSHV and EBV co-infected B cells in mouse models point to a role for EBV in PEL pathobiology. To test whether the presence or absence of EBV is a major determinant of genetic requirements, we performed principal component analysis (PCA) on normalized sgRNA counts (Fig. 1f). Using this unbiased approach, PEL cell lines did not cluster based on their EBV infection status. Analyses of other principal components (up to PC5) were similarly unable to separate cell lines based on EBV infection status. These results indicate that EBV(−) and EBV(+) PEL cell lines in general have highly similar genetic requirements for their survival.

However, it remains possible that individual genes are selectively needed in EBV(−) or EBV(+) PEL cell lines, to either compensate for the absence of EBV or to facilitate latent maintenance of two herpesviruses. A small number of differentially required genes would be missed in the global unsupervised clustering analyses above. Indeed, we identified 35 candidates for genes that preferentially scored as dependencies in EBV(−) PEL cell lines (Supplementary Figure 2). In contrast, only three gene dependencies were preferentially detected in the majority of EBV(+) cell lines. Individual candidates for differential requirements based on EBV co-infection thus exist and should be investigated in future studies. Overall, however, our CRISPR screens suggest that EBV(−) and EBV(+) PEL are a single disease driven by a common set of oncogenic addictions.

### The oncogenic landscape of PEL

**Fig. 2:**
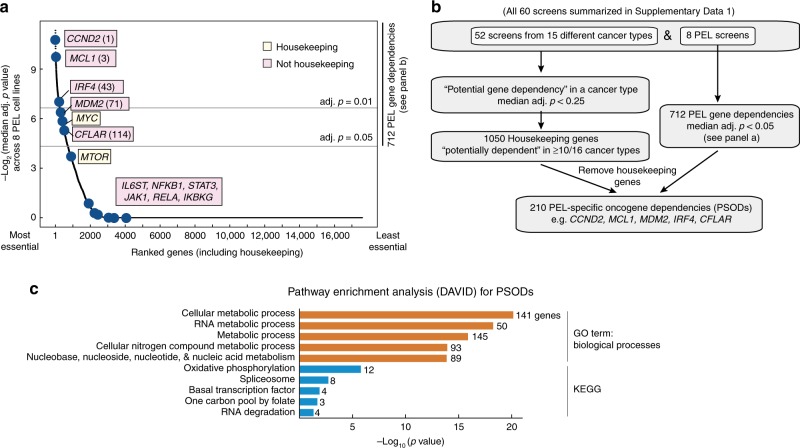
Genetic dependencies of PEL cell lines. **a** Significance of dependency of all genes screened by Brunello library in 8 PEL cell lines. Genes are ranked using the median adj. *p* value scores (FDR). A large majority of genes, including those involved in NF-κB (e.g., *RELA*, *NFKB1*, and *IKBKG*) and cytokine signaling (e.g., *STAT3* and *JAK1*), score as dispensable in PEL cells. Genes in yellow are considered housekeeping genes, non-housekeeping genes are in pink. Ranks among PSODs are in parentheses. **b** Workflow and criteria for classifying “housekeeping genes” and “PEL gene dependencies”, based on CRISPR screens in this study and 52 publicly available screens. PEL gene dependencies that do not have housekeeping functions are further considered “PEL-specific oncogene dependencies” (PSODs). **c** Pathway enrichment analysis of PSODs using DAVID for gene sets from GO (orange) or KEGG (blue). Number of genes included in each enriched pathway is indicated. Full results are in Supplementary Data 11

To pinpoint the most critical single-gene dependencies of PEL cells, we ranked genes using their median adj. *p* value of sgRNA depletion across all eight cell lines (Supplementary Data 8). This approach defines a core set of 712 “PEL gene dependencies” (using a median adj. *p* < 0.05 cutoff; Fig. 2a). Importantly, these genes are required in all or nearly all PEL cell lines, independently of EBV co-infection, genetic background, or patient treatment history. Among the top ranked genes on this list is *CCND2*, which encodes the G1/S-specific cyclin D2 (Fig. 2a). Other top ranked genes are *MCL1* and *CFLAR*, which encode the anti-apoptotic proteins MCL1 and c-FLIP, respectively. Dependencies of PEL cell lines on cyclin D2 and c-FLIP have not been reported previously, while a potential dependency on MCL1 was suggested in recent studies^45,46^. Our screens furthermore revealed a novel dependency on *MDM2*, which encodes a negative regulator of p53 and p73. This requirement for *MDM2* by PEL is consistent with previous studies demonstrating sensitivity of PEL cell lines to re-activation of p53/p73 by the MDM2 inhibitor Nutlin-3a^9,17^. Moreover, *IRF4* scores as essential in seven of eight PEL cell lines, confirming a recent report^34^.

The 712 PEL gene dependencies above include many genes with housekeeping functions. To distinguish between housekeeping genes and those that are specifically required in PEL, we analyzed a total of 60 CRISPR screens representing 16 different cancer cell types^40–43^ (Fig. 2b and Supplementary Data 1). Genes that were depleted (median adj. *p* < 0.25) in the majority of cell lines per cancer type were classified as a “potential gene dependency” in this cancer type. Genes that were “potential gene dependencies” in ≥10/16 cancer cell types were further considered “housekeeping genes.” These cutoffs were chosen to account for false negatives and flag 1050 genes as housekeeping genes (Fig. 2b and Supplementary Data 9). Removing these genes from the list of PEL gene dependencies identifies 210 genes that are specifically required in PEL but are unlikely to be housekeeping genes. We refer to these 210 genes as “PEL-specific oncogenic dependencies” (PSODs, Supplementary Data 10). PSODs are expected to include the main PEL-specific oncogenic drivers. PSODs also likely include genes that are essential for the episomal maintenance of the KSHV genome and/or to prevent lytic re-activation. Because viral latency is necessary for PEL, such genes can be considered non-traditional oncogenes in the broadest sense.

Previous reports suggest that PEL cells are addicted to overexpression of MYC and high levels of mTOR activity^26^. However, these two genes are not classified as PSODs, because both are flagged as housekeeping genes (Fig. 2a and Supplementary Data 9). CRISPR/Cas9 editing results in complete inactivation and thus these screens cannot distinguish addictions to overexpression or constitutive activation of genes from their housekeeping functions.

Pathway analysis of the PSODs showed enrichment in pathways involved in several metabolic processes (Fig. 2c and Supplementary Data 11). These enrichments could point to specific metabolic demands of PEL cells, an idea that is supported by an emerging literature on how KSHV reshapes the metabolic status of infected cells^47^.

Strikingly, the list of PSODs included several genes that can be inhibited by compounds either in pre-clinical development or already in clinical use for other cancers, such as *CCND2*, *IRF4*, *MDM2*, and *MCL1*. PEL is a very rare disease, which complicates clinical trial design. Thus, repurposing already available drugs is likely the most practical option for treatment strategies in PEL. These genes were therefore further investigated below.

In sum, we identify a set of 210 genes that are specifically required in PEL cell lines (defined as PSODs). Because these genes do not have housekeeping functions, they represent attractive therapeutic targets.

### Single oncogene dependencies of PEL cells on *IRF4* and *MDM2*

**Fig. 3:**
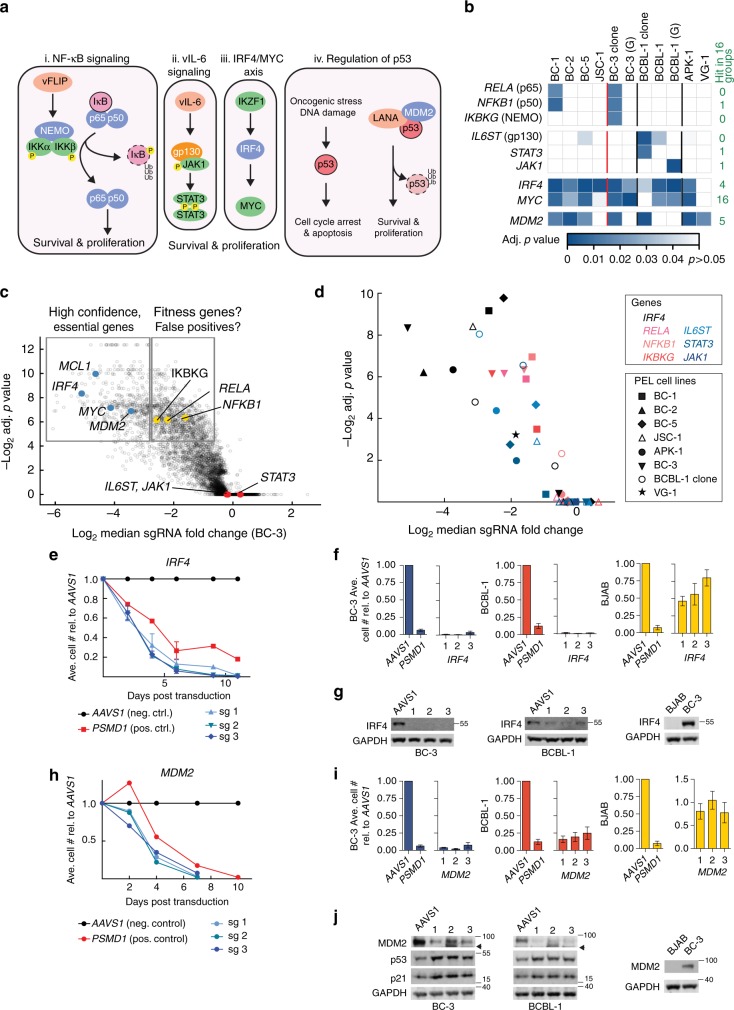
PEL cell lines depend on *IRF4* and *MDM2*, but not NF-κB components. **a** Current models of NF-κB, vIL-6, IRF4/MYC axis, and p53 regulatory pathways in PEL. i In the inactive state, the NF-κB subunits p65 and p50 are sequestered by the IκB complex and prevented from signaling. Upon activation of the pathway, the IKK complex (NEMO, IKKα, and IKKβ) is phosphorylated and targets IκB for degradation. This releases the p65/p50. In PEL, this pathway is thought to be constitutively activated by interaction of vFLIP with NEMO (*IKBKG*). ii Autocrine signaling by vIL-6 is triggered by the intracellular binding to gp130, which subsequently activates JAK/STAT signaling. iii The IRF4/MYC axis is proposed as a pro-proliferative transcriptional axis downstream of IKZF1. iv Activity of the tumor suppressor p53 in PEL is blocked by its degradation via the LANA-MDM2 complex. Genes in blue were chosen for validation. **b** Heatmap of adj. *p* values of sgRNA of key genes from **a** across cell lines screened. On the right are the numbers out of 16 cancer types where the relevant gene scored with a median adj. *p* < 0.25 in each group (Fig. 2b). The Brunello library was used for most of the screens except where indicated: G, GeCKO v2. **c** Volcano plot for genes screened using Brunello library in BC-3 highlighting some high confidence PEL dependencies (blue), fitness genes (yellow), and dispensable genes (red). **d** Degree of depletion of NF-κB genes (pink), genes that are involved in vIL-6 signaling (blue), and *IRF4* (black) in all PEL cell lines screened by the Brunello library. **e** Representative analysis of relative live cell numbers over time after IRF4 knockout in BC-3 cells, see Supplementary Figure 4 for details. **f** End-point analysis of several independent growth curves (as in **e**) for *IRF4* knockout in Cas9-expressing BC-3, BCBL-1, or BJAB cell clones. **g** Representative western blots of cells in **f**. **h**–**j** Similar to **e**–**g** but following *MDM2* knockout. Arrowhead, truncated MDM2 from CRISPR targeting. AAVS1, control sgRNA targeting the non-coding *AAVS1* locus; PSMD1, sgRNA targeting the housekeeping gene *PSMD1*. Error bars represent SEM, *n* ≥ 3

Surprisingly, neither the PEL gene dependencies nor PSODs include key genes involved in the NF-κB (e.g., *RELA*, *NFKB1*, and *IKBKG*) and cytokine signaling pathways (e.g., the v-IL6 receptor *IL6ST* and JAK/STAT family proteins). These pathways are currently considered critical in PEL, but the relevant genes scored in only a subset of cell lines (Figs. 2a and 3a, b). Even in these cases, the sgRNAs targeting these genes were only modestly depleted (Fig. 3c, d). These results could thus be false negative or these genes could serve as “fitness genes,” which provide a subtle advantage to at least a subset of PEL cell lines.

These unexpected results prompted us to establish a robust workflow for the validation of individual PSODs. To achieve highest sensitivity, validation was done in clonal BC-3 and BCBL-1 Cas9 cell lines that display optimal and consistent gene editing^48^ and retain their ability to undergo lytic re-activation (Supplementary Figure 3a). Parallel genome-wide screens done with a BCBL-1 Cas9 cell pool and a matched clone had overall similar results, indicating that the clone remains functionally similar to the parental cell pool (Supplementary Figure 3b). However, superior editing efficiencies in cell clones resulted in increased sensitivity and the identification of an extended number of significantly depleted genes, likely fitness genes (Fig. 1c). Using these cell lines, we individually targeted genes for functional knockout following lentiviral sgRNA transduction (Supplementary Figure 4). sgRNAs against the non-coding *AAVS1* locus^49^ and the essential proteasome subunit *PSMD1*^48^ served as negative and positive controls, respectively. Following sgRNA transduction, absolute live cell counts were monitored over time using flow cytometry.

We first examined the dependency of PEL cells on *IRF4*, which scored highly in seven of eight screens and had previously been shown to be essential in the PEL cell line BC-3 using RNA interference^34^ (Figs. 2a and 3a, b). IRF4 is furthermore downregulated by treatment of PEL cell lines with IMiDs, suggesting that a dependency on IRF4 in PEL cells may be druggable. Targeting *IRF4* by three independent sgRNAs resulted in a rapid and complete loss of viability in BC-3 and BCBL-1 (Fig. 3e–g). These effects were not seen in the KSHV-negative B cell line BJAB, where IRF4 is not expressed (Fig. 3f, g). These data therefore confirm that *IRF4* is indeed among the most strongly and specifically required cellular genes in PEL cell lines.

We next targeted MDM2, an oncogenic E3 ubiquitin ligase that triggers degradation of the tumor suppressor p53 family of proteins (Fig. 3a). Like *IRF4*, sgRNAs for *MDM2* were strongly depleted in most PEL screens (Fig. 3b). A role for MDM2 in the survival of PEL cells had been strongly suggested by its pharmacological inhibition using Nutlin-3a, but has not been demonstrated directly^9,17^. sgRNAs for *MDM2* triggered rapid cell death in BC-3 and BCBL-1 (Fig. 3h–j). Loss of MDM2 resulted in the expected stabilization of p53 and consequent upregulation of the p53 target p21 (Fig. 3j). As in the case of *IRF4*, targeting *MDM2* in BJAB, which do not express MDM2, did not affect cell viability (Fig. 3i, j). Taken together, this is the first direct demonstration that PEL cell lines critically require *MDM2*, despite the p53 inhibitory activities of LANA and vIRF3.

Having successfully validated requirements for *IRF4* and *MDM2*, we individually inactivated *RELA*, *NFKB1*, and *IKBKG*, as examples for genes that unexpectedly did not score as essential in most screens. These experiments confirmed that these genes are indeed dispensable in BC-3 and BCBL-1 at least in vitro (Supplementary Figure 5). Because these genes score as a group in the BC-3 and BC-1 screens (Fig. 3b and Supplementary Data 8), a subtle fitness function in at least these two cell lines appears likely. We note that our validation setup may not have the sensitivity to robustly detect fitness effects of less than two- threefold cumulative reductions in live cell numbers at the end of ~2- to 3-week growth curves, which are expected to reach statistical significance in pooled screens. In sum, our results strongly suggest that the role of genes in the NF-κB and vIL-6 signaling pathways in cultured PEL cell lines may be surprisingly subtle and should be re-evaluated (see Discussion). Our screens and validation experiments on the other hand, demonstrate critical requirements for *IRF4* and *MDM2* in all PEL cell lines.

### PEL cells require cyclin D2 and c-FLIP expression

**Fig. 4:**
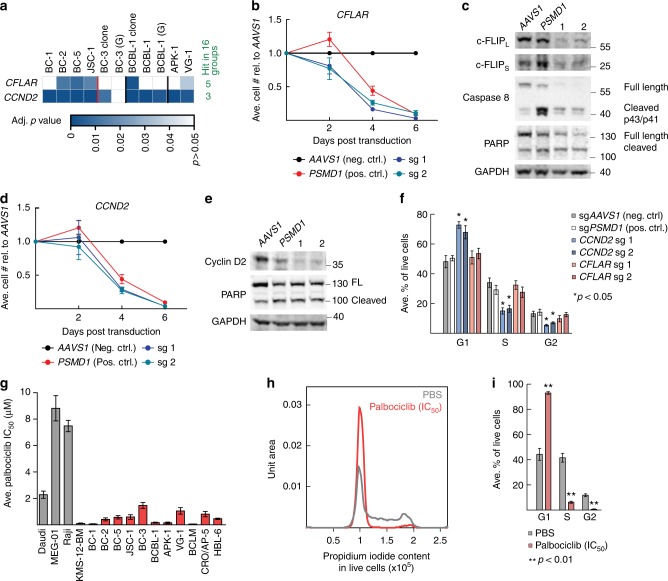
PEL cell lines are dependent on *CCND2* and *CFLAR*. **a** Heatmap of adj. *p* values of sgRNA depletion of *CCND2* and *CFLAR* in cell lines screened. Indicated on the right are the numbers of cancer types (out of 16) where the gene was found to be a potential dependency. The Brunello library was used for most of the screens except where indicated: G, GeCKO v2. **b**, **c** Knockout of *CFLAR*. **b** Representative analysis of relative live BCBL-1 cell numbers over time following *CFLAR* knockout. *n* = 4. **c** Representative western blots of c-FLIP_L_ and c-FLIP_S_ isoforms for **b**. **d**, **e** Similar to **b** and **c** but using *CCND2* sgRNAs. Experiments in **b**–**e** were performed together and thus share controls. **f** Distribution of cell cycle phase populations in BCBL-1 Cas9 cells upon *CCND2* or *CFLAR* knockout analyzed by propidium iodide staining of samples on day 4 in experiments shown in **b**–**e**. *p* Values were calculated by Student’s *t* test and compared to sgAAVS1. **g** Calculated IC_50_ values of palbociclib in the indicated cell lines. Gray bars, non-PEL cells; pink bars, PEL cell lines; *n* ≥ 3. **h**, **i** Cell cycle analysis of propidium iodide-stained live BCBL-1 cells treated for 24 h with 220 nM palbociclib (IC_50_). **h** Representative histograms of DNA content from propidium iodide staining. **i** Distribution of cell cycle phase populations. *p* Values were calculated by Student’s *t* test and compared to PBS-treated cells. *n* = 3. All error bars, SEM

Our screens identified *CCND2*, encoding cyclin D2, as the top ranked PSOD in PEL cell lines (Figs. 2a and 4a). This dependency was surprising, given that PEL cell lines express vCYC. Similarly, *CFLAR*, which encodes c-FLIP, confidently scored as a PSOD despite expression of its viral homolog vFLIP (Figs. 2a and 4a). c-FLIP functions to block FADD-mediated apoptosis by preventing the activation of the initiator pro-caspase 8, among several other roles. vCYC and vFLIP are sufficiently distinct from their cellular counterparts to exclude cross-inhibition by sgRNAs targeting *CCND2* or *CFLAR*.

Targeting *CFLAR* for knockout resulted in a rapid decrease in live BCBL-1 cell numbers (Fig. 4b). This is accompanied by a cleavage of pro-caspase 8 and PARP (Fig. 4c), suggesting that c-FLIP is required to block apoptosis in BCBL-1. Similarly, inactivation of *CCND2* in BCBL-1 cells led to a rapid reduction of live cell numbers (Fig. 4d, e). As expected, loss of cyclin D2, but not c-FLIP, led to cell cycle arrest, as indicated by the accumulation of cells at the G1 phase of the cell cycle and a corresponding decrease in the percentage of cells in the S and G2 phases (Fig. 4f). However, we note that loss of cyclin D2 eventually triggered apoptosis in BCBL-1, as indicated by PARP cleavage (Fig. 4e).

This selective and strong dependency of PEL cells on *CCND2* could be exploited as a therapeutic strategy. Palbociclib is a clinically-approved inhibitor of CDK4 and 6 currently used for treating ER^+^HER2^−^ breast cancers. Since D-type cyclins function by binding to CDK4/6, palbociclib also inhibits cyclin D activity. Indeed, 11 tested PEL cell lines were highly sensitive to palbociclib with IC_50_s ranging from 73 nM to 1.5 µM (Fig. 4g). The inhibitor was similarly effective against KMS-12-BM, which depends on cyclin D1 overexpression^50^. As expected, pablociclib treatment of PEL cell lines lead to a striking G1 arrest, which is almost complete in BCBL-1 cells (Fig. 4h, i, and Supplementary Figure 6 for results in BC-3).

Our data collectively show for the first time that PEL cell lines are surprisingly dependent on c-FLIP and cyclin D2, which is druggable. Palbociclib could in principle function by affecting the function of cyclin D2 and/or vCYC in PEL. Regardless, this drug offers a promising novel treatment strategy for PEL. Furthermore, our findings demonstrate that vCYC and vFLIP cannot compensate for the loss of their cellular counterparts.

### MCL1 is critical in PEL cells

**Fig. 5:**
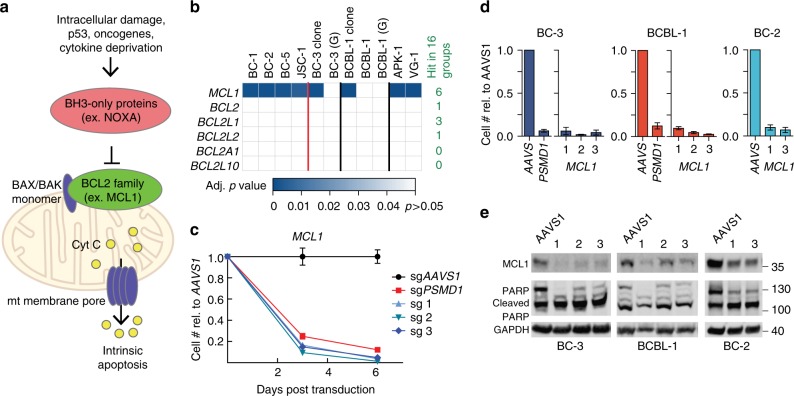
PEL cell lines are addicted to MCL1. **a** BCL2 family proteins primarily function on the outside of the mitochondrial membrane to prevent BAX or BAK monomers from oligomerizing to form outer membrane pores. Upon intracellular stress, pro-apoptotic BH3-only proteins are upregulated and bind to the BCL2 proteins, thereby competing with BAX or BAK. The free BAX or BAK monomers then oligomerize to form outer mitochondrial membrane pores, resulting in cytochrome c to the cytosol and initiation of apoptosis. **b** Heatmap of adj. *p* values of sgRNA depletion of the BCL2 family genes across screens. Indicated on the right are the numbers of cancer types (out of 16) where the gene was found to be potentially essential. The Brunello library was used for most of the screens except where indicated: G, GeCKO v2. **c** Representative growth curve analysis following *MCL1* knockout in BC-3 Cas9 (*n* = 3, technical replicates). **d** End-point analysis of several growth curves for *MCL1* knockout in BC-3, BCBL-1, or BC-2 Cas9 cells (*n* = 3, biological replicates). **e** Representative western blots for *MCL1* knockout and PARP cleavage for experiments in **d**. All error bars, SEM

*MCL1* ranked as the third highest PSOD across all PEL cell lines. MCL1 is a member of the anti-apoptotic BCL2 family of proteins, which prevent the formation of outer mitochondrial membrane pore channels by BAX and BAK (Fig. 5a)^51^. Outer mitochondrial membrane permeabilization in turn triggers apoptosis via the intrinsic pathway. Strikingly, of all the BCL2 family members, only *MCL1* showed a strong and consistent requirement in all PEL cell lines (Fig. 5b and Supplementary Figure 7). Overexpression of MCL1 by gene amplification has been observed in a diverse range of cancers^52^. Importantly, MCL1 was recently shown to be susceptible to direct and specific inhibition by the small molecule S63845 in other hematological malignancies^53^. Previous reports have pointed to a possible dependency on *MCL1* of PEL cells. BH3-profiling of BCBL-1 indicated a hybrid MCL1 signature that was initially attributed to the expression of the viral BCL2 homolog, a lytic protein^45^. Treatment with a HSP90 inhibitor PU-H71 induces massive apoptosis in PEL cells by destabilizing HSP90 clients, including MCL1^46^. PU-H71 furthermore synergizes with a pan-BCL2 inhibitor that also inhibits MCL1. However, neither study directly assessed the contribution of MCL1 to the survival of PEL cell lines.

A vital role for MCL1 in PEL cells is confirmed by a dramatic loss of cell viability as early as 3 days following *MCL1* sgRNA transduction of BC-3 (Fig. 5c–e). Similarly, striking dependencies on MCL1 were also seen in the PEL cell lines BCBL-1 and BC-2 (Fig. 5d, e). PARP westerns confirmed that loss of MCL1 is a highly efficient trigger of apoptosis in PEL cell lines, as expected (Fig. 5e). The dependency on high levels of MCL1 expression is furthermore validated in a panel of PEL cell lines using short hairpin RNA (shRNA)-mediated knockdown (Supplementary Figure 8). All cell lines, except for BC-2, were highly sensitive to even modest shRNA-induced reductions of MCL1 expression and underwent apoptosis. The lack of a response in BC-2 is likely due to the marginal knockdown of MCL1 in this cell line, because *MCL1*-specific sgRNAs resulted in rapid cell death in BC-2 (Fig. 5d, e). Taken together, our findings demonstrate that PEL cell lines strongly depend on MCL1 oncogene addiction for their survival.

### MCL1 is a candidate drug target in PEL

**Fig. 6:**
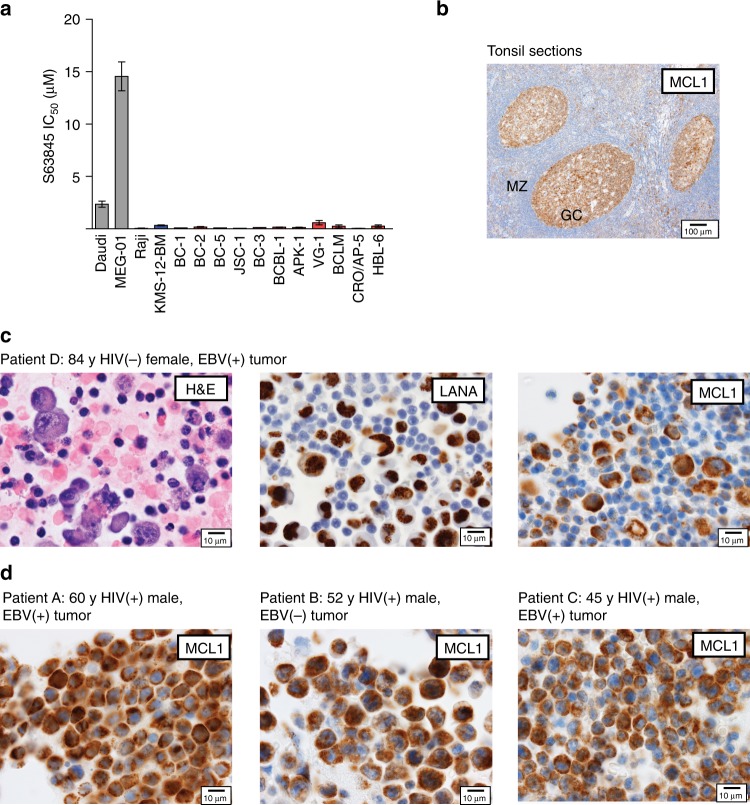
Pharmacological inhibition of MCL1 in PEL and control cell lines and MCL1 expression in PEL tumors. **a** Calculated IC_50_ values of S63845 in different cell lines. Based on Kotschy et al.^53^, Daudi and MEG-01 cells are MCL1-independent cell lines while Raji and KMS-12-BM are MCL1-dependent cell lines. Error bars, SEM; *n* ≥ 3 biological replicates. **b** MCL1 staining in tonsilar sections confirms specificity of our staining protocol. Germinal center cells (GC) express high levels of MCL1 while mantle zone cells (MZ) express low to undetectable levels of MCL1. **c** Example of a histological characterization of tumor sections from patient D (84-year HIV(−) female with EBV(+) tumor) with hematoxylin and eosin staining (H&E), LANA immunohistochemical stain, or MCL1 immunohistochemical stain. **d** MCL1 immunohistochemical stains for tumor sections from patients A–C. Corresponding scale bars are depicted in lower right corner of each image

The striking and consistent addiction of PEL cells to MCL1 expression prompted us to test the therapeutic potential of the newly developed MCL1 inhibitor S63845^53^ by assessing its efficacy in a panel of 11 PEL cell lines. MCL1 inhibition by S63845 proved lethal in all tested PEL cell lines, with IC_50_ values in the nanomolar range (Fig. 6a). Notably, the response to S63845 in PEL cell lines was comparable to that seen in the MCL1-dependent B cell lines Raji and KMS-12-BM, which were tested in the earlier report^53^. In contrast, the MCL1-independent cell lines Daudi and MEG-01 were 12- and 77-fold less sensitive to the inhibitor compared to the PEL cell lines, confirming the expected specificity of treatment^53^. In sum, we show that inhibiting MCL1 activity with S63845 is a highly effective and promising strategy that should be further developed for the treatment of PEL.

### MCL1 is highly expressed in PEL tumors

The exquisite dependence of PEL cell lines on MCL1 and the feasibility of its therapeutic targeting by S63845 led us to examine the relevance of this oncogene in PEL tumors. Immunohistochemistry specifically detected high levels of MCL1 in germinal centers but not in marginal zones of tonsillar sections, as expected^54^ (Fig. 6b). Importantly, tumor samples from four independent PEL cases at the Northwestern Memorial Hospital since 2010 showed very high expression of MCL1 specifically in the KSHV-infected tumor cells (Fig. 6c, d). High levels of MCL1 expression in PEL tumor cells further support its oncogenic role and viability as a drug target in PEL.

## Discussion

**Fig. 7:**
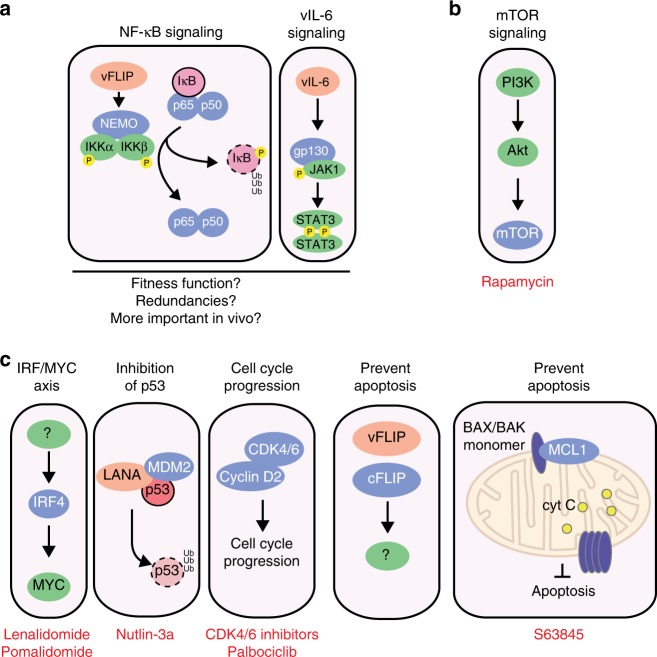
Revised working model of the main host oncogenic gene dependencies in PEL cell lines. **a** Genes involved in NF-κB and Jak/Stat signaling are dispensable in most PEL cell lines and may serve fitness functions. **b** Addiction to constitutively active mTOR signaling was not captured in this study, due to confounding housekeeping function of mTOR. **c** Critical PEL-specific oncogene dependencies (PSODs) on *IRF4*, *MDM2*, *CCND2*, *CFLAR*, and *MCL1* (in blue), most of which are druggable using agents shown in red

The oncogenic mechanisms underlying PEL are poorly understood and this disease consequently remains largely incurable. Here we utilized genome-wide loss-of-function CRISPR screens to reveal genetic dependencies of eight PEL cell lines. Comparative analysis of similar datasets from 16 different types of cancer cell lines, including a newly generated dataset from multiple myeloma, allowed us to discover 210 non-housekeeping single-gene dependencies of PEL (PSODs). We validate several of these novel dependencies and reveal cyclin D2 and MCL1 as attractive candidates for drug targets in PEL. This work thus serves as an unbiased and comprehensive resource for human genes that are critical in PEL cell lines and points to novel strategies for therapeutic intervention in this aggressive lymphoma. A revised working model of PEL biology is presented in Fig. 7.

PEL cell lines co-infected with EBV exhibited a similar set of essential gene dependencies for survival as EBV(−) PEL. Consistent with this observation, only one of four gene expression profiling studies reported a separation of PEL cell lines based on EBV co-infection^12,30–32^. This same report identified only 40 differentially expressed genes between the two groups^30^, none of which scored differentially in our screens. Thus, EBV(+) and EBV(−) PEL cell lines overall exhibit largely similar transcriptomes and similar genetic requirements for survival in culture. This finding may reflect the dominant role of KSHV in PEL and/or convergent oncogenic mechanisms in EBV(+) and EBV(−) PEL cell lines. Our findings do not rule out a role for EBV in PEL pathogenesis and in EBV(+) PEL cell lines in vitro. Indeed, our study identified a small number of candidates for genes that may exhibit differential requirements based on EBV status. Overall, the role of EBV in PEL lymphomagenesis and established PEL cell lines will require additional investigation. Future studies should also specifically address a requirement for EBV in EBV(+) PEL cell lines, by antagonizing EBV genome maintenance.

Our screens and validation experiments surprisingly suggest that key genes of the NF-κB signaling pathway are not individually essential in PEL, but perhaps act as fitness genes in a subset of cell lines (BC-1 and BC-3; Fig. 7). The current assumption that all PEL cell lines are addicted to constitutive NF-κB signaling stems from inhibitor studies using Bay 11-7082^20,55^. Multiple studies since then have demonstrated NF-κB-independent toxicity of Bay 11-7082 in different cancer cell types^56–58^. The efficacy of Bay 11-7082 against PEL cell lines may thus be due to pleiotropic effects and not solely due to inhibition of NF-κB signaling. Given that genes in the NF-κB pathway are individually non-essential in PEL cell lines, it is unlikely that this pathway controls the expression of the highly essential oncogenes *IRF4* and *MCL1* in PEL cells, although this has been observed in other cancers^59,60^. A potential role for NF-κB in PEL was also postulated based on abundant evidence that ectopic expression of vFLIP can activate this pathway via an interaction with NEMO and the finding that vFLIP is essential in PEL cell lines^13,14,20–24^. RNAi of vFLIP in the PEL cell line BC-3 has been shown to dampen NF-κB reporter activity^14^. However, these reports do not directly show that the activation of NF-κB indeed underlies vFLIP essentiality in PEL cell lines and relevant human genes have not previously been targeted for functional knockdown in PEL cells. Our screens and validation experiments show that *IKBKG* is dispensable in a large majority of PEL cell lines. While the interaction between vFLIP and NEMO is well documented, it remains possible that essential functions of vFLIP in PEL cell lines are independent of this interaction and NF-κB activation. Importantly, vFLIP is also known to inhibit autophagy and death receptor signaling^22,61^ and is important for c-FLIP expression, at least in BC-3^14^. Moreover, the strong requirement of PEL cell lines for c-FLIP and cyclin D2 reveals that expression of vFLIP and vCYC in PEL cell lines cannot compensate for the loss of these cellular proteins. In principle, vFLIP and vCYC expression could serve to overexpress c-FLIP and cyclin D2-like functions. On the other hand, it is possible that vFLIP and vCYC have independent and non-redundant functions. One important caveat of our approach is that we employ single-gene knockouts to interrogate gene dependencies. We therefore cannot rule out compensating mechanisms, such as the non-canonical arm of the NF-κB signaling pathway or redundancies between the NF-κB and cytokine signaling pathways. Lastly, these pathways could have more relevant roles in vivo and become individually non-essential during adaptation to cell culture. Indeed, a primary PEL tumor was reported to have dramatically higher levels of active nuclear NF-κB than the BC-5 cell line, which was derived from the same tumor^20^. Because the manipulation of NF-κB signaling by KSHV gene products is well established, the relevance of this pathway to lymphomagenesis should be carefully addressed using non-heterologous systems, such as PEL tumor samples and patient derived xenografts, which is beyond the scope of this study.

A practical utility of our study is the discovery of drug targets that can be further developed for therapeutic intervention in PEL (Fig. 7). We present the first direct evidence for a key role of *MDM2* in PEL cells, providing a genetic basis for the use of its inhibitor Nutlin-3a^9,17,18,62^ as a candidate therapeutic strategy. Moreover, our study strongly supports a critical role of IRF4 addiction in PEL cells. Our data thus strengthen the rationale for the development of IMiDs, which have been shown to trigger loss of IRF4 expression in PEL^34^, as a treatment strategy. In line with this, lenalidomide is currently in clinical trials for classical and extra-cavitary PEL^63^. We note, however, that our screens and validation experiments failed to support a recently reported role of IKZF1 upstream of IRF4 in PEL^64^. Because IKZF1 has recently been reported as the relevant IMiD effector in PEL, the mechanisms of action of IMiDs in PEL require further investigation.

Importantly, we identified novel druggable dependencies of PEL cell lines on cyclin D2 and MCL1. While palbociclib is highly effective in controlling proliferation in culture, cell cycle arrest in PEL cells eventually triggers cell death, making palbociclib a promising treatment strategy. S63845, on the other hand, exerts a direct cytotoxic effect—by directly neutralizing MCL1 and activating apoptosis in MCL1-dependent tumors in culture and in vivo in other cancers^53^. At least BC-3 is weakly dependent on the BCL2 family member *BCL2L1* (encoding Bcl-xL; Supplementary Figure 7). However, BC-3 cells were just as sensitive to S63845 and MCL1 knockdown as MCL1-only-dependent PEL cell lines. Altogether, the highly selective addiction to MCL1 and the high sensitivity of PEL cell lines to even a partial decrease in its expression makes MCL1 an appealing and viable strategy for therapeutic intervention.

In conclusion, we utilized genome-wide CRISPR screens to identify PEL core dependencies that serve as effective druggable genetic vulnerabilities. Importantly, repurposing of existing inhibitors for PSODs could significantly alleviate the difficulty in developing drugs and designing clinical trials for this exceedingly rare cancer. We anticipate that our study will establish a framework for future studies on PEL transformation, KSHV biology, and inspire the development of much needed therapeutic interventions.

## Methods

### Cells

HEK-293T cells were cultured in Dulbecco’s modified Eagle’s medium (Lonza, Walkersville, MD), supplemented with 10% fetal bovine serum (FBS; Corning, Manassas, VA) and 10 µg/mL gentamycin (VWR, Radnor, PA). Most suspension cells (APK-1, BC-2, BC-3, BC-5, VG-1, CRO/AP5, BCLM, HBL-6, KMS-12-BM, and MEG-01) were maintained in RPMI-1640 (Lonza) containing 20% FBS or Serum Plus-II (Sigma), 10 µg/mL gentamycin, and 0.05 mM β-mercaptoethanol (Bio-Rad, Hercules, CA). The following cell lines were grown in similar medium but were supplemented with 10% FBS or Serum Plus Medium: BJAB, BC-1, JSC-1, BCBL-1, Raji, and Daudi. All suspension cell lines were maintained at concentrations between 2 × 10^5^ and 12 × 10^5^ cells/mL during routine culture and experiments, and were routinely tested for potential mycoplasma contamination. BC-1, BC-2, BC-3, BCBL-1, KMS-12-BM, and BJAB were validated by short tandem repeat (STR) profiling through the Northwestern University NUSeq core facility. For other PEL cell lines reference STR profiles are not available and these cell lines were therefore validated by PCR for KSHV or EBV infection status.

### CRISPR library preparation in *Escherichia coli*

GeCKO v2 libraries A and B^38,65^ and Brunello library^37^ were obtained from Addgene. The GeCKO v2 libraries A and B contain 122 411 unique sgRNAs that target 19 050 human genes and 1864 miRNAs. The inclusion of 6 sgRNAs for each coding gene and of 4 sgRNAs for each miRNA accounts for potential off-target effects of individual sgRNAs. miRNA data are not reported here due to several confounding caveats, such as overlapping essential genes and the less straight forward targeting of non-coding RNAs using CRISPR/Cas9. The Brunello library was designed to target 19 114 human genes with 76 411 sgRNAs (4 sgRNAs/gene). Libraries were transformed separately into Endura ElectroCompetent *E. coli* cells (Lucigen, Middleton, WI) in four electroporation reactions, each containing 25 µL bacteria and 100 ng plasmid library. Electroporations were performed in 1 mm cuvettes (VWR) using an exponential decay pulse (10 µF, 600 Ω, 1800 V) with a Gene Pulser Xcell (Bio-Rad). Each reaction was immediately cultured in a 17 × 100 mm tube containing 2 mL Recovery Medium (Lucigen) for 1 h at 37 °C on a bacterial shaker at 250 rpm. Broth cultures from each library were pooled, plated onto 20 LB-ampicillin plates, and grown for 14–15 h at 32 °C. This yielded >1500-fold sgRNA representation based on colony numbers obtained from serial dilutions. Bacterial cells were harvested directly from plates and plasmid DNA was extracted over four columns of PerfectPrep Endofree Maxi Kit (5 Prime, Hilden, Germany).

### Cloning of individual sgRNA constructs

All sgRNAs were cloned into plentiGuide-Puro^38^ using the BsmBI cloning site (New England Biolabs, Ipswich, MA). sgRNA sequences targeting specific genes were designed using publicly available tools^37,66^ or were derived from the Brunello library. sgRNA sequences and sequences of primers used for cloning are listed in Supplementary Data 12.

### Lentivirus preparation

For library production, 10^7^ HEK-293T cells were seeded per 15 cm dish the day before transfection. Plasmids expressing Cas9 or sgRNAs (11 µg) were co-transfected with 5.48 µg pMD2.G and 8.24 µg psPAX2 using 158 µL of a 15.6 mM solution of Polyethylenimine HCl MAX (Linear, MW 40,000, Polysciences, Warrington, PA). For the co-transfection of the GeCKO libraries, 5.5 µg each of Libraries A and B were added per transfection reaction. For the Brunello library, a total of 11 µg was used per 15 cm dish. For individual sgRNA lentivirus production, reactions were scaled down based on relative surface area where needed. After 6–8 h, media were replaced with RPMI-1640 containing 20% FBS. Lentiviruses were harvested after 3 days, by centrifugation of the supernatant at 1200 × *g* for 8 min and filtration through a 0.45 µm membrane. Viral titers were determined in naive cells by infecting these cell lines with increasing amounts of lentiviruses in the presence of 4 µg/mL polybrene. After 1 day, 1 µg/mL puromycin (for sgRNA and library lentiviruses) or 10 µg/mL blasticidin (for Cas9 viruses) was added. Live cell numbers were determined by trypan blue exclusion after 2 (puromycin selection) or 3 days (blasticidin selection) and used to calculate viral titers.

### Cas9-expressing cell lines

B cells (2–5 × 10^5^ cells/mL) were transduced with lentiCas9-Blast virus^38^ at MOIs between 0.7 and 1.5 in the presence of 4 µg/mL polybrene. The next day, the medium was replaced and supplemented with 10 µg/mL blasticidin. Selection was continued for 3–5 days until no viable cells remained in an untransduced control plate treated with blasticidin. Pooled Cas9 cells were expanded under blasticidin selection up to their transduction with the CRISPR libraries.

Clonal Cas9-expressing B cells were derived from pooled Cas9 cells using limiting dilution cloning into round bottom 96-well plates for 2–3 weeks under blasticidin selection. Resulting clones were screened for optimal Cas9-Flag expression by anti-Flag M2 immunoblotting (Sigma-Aldrich, St. Louis, MO). Clones expressing the highest Cas9 levels were functionally screened for editing efficiency following transduction with a negative control sgRNA AAVS1 or a positive control sgRNA against the housekeeping gene *PSMD1* as previously reported^48^. Cell viability was monitored for 7–10 days. Clones that resulted in robust cell death upon transduction with PSMD1 sgRNA were considered optimal for Cas9-mediated editing and used for subsequent validation experiments.

### CRISPR screens

Dropout screens were performed similarly to Sanjana et al.^38^. B cell pools expressing Cas9 were produced as described above. Cell pools were seeded in eight 15 cm dishes per replicate at a density of 7.5 × 10^5^ cells/mL in 50 mL complete medium per dish and infected with GeCKO or Brunello library viruses at an multiplicity of infection (MOI) of 0.3 in the presence of polybrene (4 µg/mL). Three replicate infections were set up per cell line. Cell numbers were chosen to allow for a theoretical ~500× representation per sgRNA and replicate.

After 24 h, cells were recovered by centrifugation and plated in fresh media containing puromycin (1 µg/mL) to select for library transduced cells. A matched plate with untransduced cells was similarly put under selection. After 2–3 days, or when no viable cells remained in the control plate, cells were washed with phosphate-buffered saline (PBS) and seeded in fresh media without antibiotics in five 15 cm plates per replicate, at 3 × 10^5^ cells/mL in 50 mL/plate. Leftover cells were pelleted and snap-frozen. These samples were used for “input” DNA library preparation for the GeCKO libraries.

Cell numbers were monitored every 2 days and re-adjusted to 3 × 10^5^ cells/mL in 50 mL in each of five 15 cm plates per replicate. This approach maintains a theoretical ~500× coverage for each sgRNA. At days 14–18, surviving cells were washed with PBS, collected, and cell pellets were snap-frozen.

### Purification of genomic DNA

Genomic DNA was purified from frozen cell pellets following a published protocol^67^. Working on ice, 0.15–0.2 g of cell pellet was thawed, and lysed in 6 mL NK lysis buffer (50 mM Tris, 50 mM EDTA, and 1% SDS, pH 8) containing 30 µL of 20 mg/mL proteinase K (Roche, Penzberg, Germany). Cell lysates were incubated for 14–16 h in a 55 °C water bath. The following day, 30 µL of 10 mg/mL RNase A (Zymo Research, Irvine, CA) was added and incubated at 37 °C for 1 h. Lysates were chilled on ice for 15 min. Proteins were precipitated by adding 2 mL pre-chilled 7.5 M ammonium acetate. The mixture was distributed over several 1.5 mL microfuge tubes and spun at 15 000 × *g* for 10 min at 4 °C. Clarified lysates were transferred to new 15 mL tubes and 0.7 volume of isopropanol was added to precipitate genomic DNA. DNA was pelleted at 2700 × *g* for 30 min at 4 °C. Genomic DNA was washed briefly with ice-cold 70% ethanol. Residual ethanol was removed and pellets were air-dried for 5 min. Purified DNA was dissolved in 1 mL Buffer EB (Qiagen, Hilden, Germany) for 1 h at 65 °C 1400 rpm. When undissolved DNA remained, solutions were placed on a rocker at 4 °C overnight. DNA preparations were stored at 4 °C.

### Library amplification and sequencing

Primer sequences for sequencing library construction were based on Sanjana et al.^38^. Primers used for library preparation were polyacrylamide gel electrophoresis-purified and synthesized by IDT (Coralville, IA).

Conditions for amplification of integrated lentivirus insert from genomic DNA were established by pilot PCR reactions to optimize amounts of genomic DNA, DNA polymerase, and number of PCR cycles for each replicate, cell line, and time point. Primer sequences are listed in Supplementary Data 13.

The final protocol used for PCR 1 for the GeCKO libraries is described below. For each 50 µL PCR reaction, 0.15 mM dNTP, 0.15 µM forward primer v2Adaptor_F^38^, 0.15 µM reverse primer R1^65^, 3 µg purified genomic DNA, 1× Q5 Reaction Buffer, and 0.5 µL Q5 High Fidelity DNA polymerase (NEB). For each replicate, a total of 133 parallel PCR reactions were performed on a total of 400 µg genomic DNA to maintain 500× coverage for sgRNA amplification. PCR conditions were as follows: initial denaturation at 98 °C for 90 s, followed by specific number of PCR cycles of 98 °C for 10 s, 60 °C for 10 s, and 72 °C for 10 s. The number of cycles, chosen based on pilot PCRs, for BC-3, BCBL-1, and BJAB were 27, 25, and 29, respectively, to result in non-saturating amplification.

The final protocol used for PCR for the Brunello libraries is described below. For each 50 µL PCR reaction, 0.15 mM dNTP, 0.13 µM forward primer v2Adaptor_F, 0.13 µM reverse primer R1_new, 6 µg purified genomic DNA, 1× Standard Taq buffer (NEB), and 0.5 µL Taq polymerase. For each replicate, a total of 42 parallel PCR reactions were performed on a total of 252 µg genomic DNA to maintain 500× coverage for sgRNA amplification. PCR conditions were as follows: initial denaturation at 95 °C for 2 min, followed by specific number of PCR cycles of 95 °C for 25 s, 55 °C for 25 s, and 68 °C for 30 s. The number of cycles, chosen based on pilot PCRs, was between 21 and 24 to result in non-saturating amplification.

The PCR 1 reactions for each replicate and time point were combined and used directly to optimize conditions for PCR 2. PCR mixes were essentially similar to PCR 1, but contained 1 µL of pooled PCR 1 products as template, and 0.15 µM each of forward and reverse primers (Supplementary Data 13). Cycling conditions were optimized for each PCR 1 product using Q5 polymerase. For the final PCR 2 amplification, a total of 6 (Brunello) or 12 (GeCKO) reactions per sample were prepared with two reactions each for a different staggered forward Illumina primer. Each replicate and time point used one barcoded reverse Illumina primer.

The PCR reactions for each PCR 2 were pooled, concentrated through a column, and resolved in 2% agarose gels. Gel slabs were ground using disposable plastic pestles and dissolved in three volumes of Buffer DF (IBI Scientific, Peosta, IA) for 5 min at room temperature on a thermomixer. PCR products were purified over two columns of a Gel Extraction kit (IBI Scientific) and eluted in a total of 40 µL water.

Purified libraries were quantified using a Qubit 2.0 Fluorometer and the Qubit dsDNA HS Assay Kit (Thermo Fisher Scientific, Waltham, MA), and quality control was performed using an Agilent 2100 Bioanalyzer and High Sensitivity DNA Kit (Agilent Technologies, Santa Clara, CA). Individual libraries were further quantified by quantitative PCR, using the KAPA Library Quantification Kit (Kapa Biosystems, Wilmington, MA). Multiplexed samples were sequenced on the Illumina HiSeq 2500 platform using 100 bp single-end reads (GeCKO) or on the HiSeq 4000 using 50 bp single-end reads.

### Processing of sequencing reads

The 5′ and 3′ adapter sequences were removed from demultiplexed sequencing reads. Remaining reads were aligned to the CRISPR library files using Bowtie. The number of reads per sgRNA were counted and summarized in a read count table. Supplementary Data 14 summarizes library statistics.

### Sliding window analysis

A sliding window method^43,68^ was used to account for the potential effects of genomic copy number on CRISPR screens identifying essential genes in cell lines. For this, CRISPR gene scores computed from normalized sgRNA counts like those seen in Aguirre et al.^40^ were used to quantify the fitness effect of gene knockout in a cell line. Briefly, under a scenario in which there are *m* total genes being screened in cell line *j*, the CRISPR gene score for gene *i* targeted by *n* sgRNAs is given by the following equation:

$${\mathrm{CRISPR}}\,{\mathrm{gene}}\, {\mathrm{score}}_{ij} = \frac{{\mathop {\sum}\nolimits_{k = 1}^n {{\mathrm{sgRNA}}\, {\mathrm{score}}_{ijk}} }}{n}$$

where sgRNA score_*ijk*_ is given as the *z*-score transformation of the log-fold change between early and late samples in cell line *j* for a sgRNA *k* targeting gene *i*:

$${\mathrm{sgRNA}}\,{\mathrm{score}}_{ijk} = {\mathrm{log}}_2\left( {\frac{{{\mathrm{late}}\,{\mathrm{count}}_{ijk}}}{{{\mathrm{early}}\,{\mathrm{count}}_{ijk}}}} \right)$$

$${\mathrm{sgRNAscore}}_{ijk} = \frac{{\mathrm{sgRNAscore}}_{ijk}-{\mathrm{AVE}}_{{\mathrm{sgRNAscor}}e_{j}}}{{\mathrm{STDEV}}_{{\mathrm{sgRNAscore}}_{j}}}$$

Next, we computed a neighborhood score for each gene by counting the number of low CRISPR scores (<5th percentile for a cell line) in the 40 gene genomic window surrounding the gene (20 “upstream genes” and 20 “downstream” genes). Genes were flagged as suspect in a cell line if their genomic neighborhood score was >12.

### Depletion and enrichment analyses

To test for significant depletion or enrichment of sgRNAs for each gene, Sliding Window Analysis-corrected read count tables from this study or published screens^40–43^ were analyzed using the Robust Rank Approach module of MAGeCK v0.5.7^39^. This program was designed to calculate both depletion and enrichment scores for genes based on median normalized counts of each sgRNA.

### Statistical analyses

GSEA^44^ was performed using the ranked depletion scores calculated by MAGeCK. Pathway enrichment analysis for the PSODs was performed using DAVID v6.7. Additional statistical tests were performed using Python, R, and GraphPad Prism using appropriate biological replicates for each test.

### Defining housekeeping genes and PSODs

To define housekeeping genes, we compared CRISPR screens from publicly available datasets^40–43^ and this study. We limited our analyses to 17 583 genes that were screened in all libraries used. We then grouped the 60 cell lines according to cancer type to create 16 groups. We considered a gene to be a potential dependency in each group if targeting sgRNAs were significantly depleted (median FDR-adj. *p* < 0.25). Finally, we classified a gene to be a “housekeeping” gene if it scored in at least 10 of 16 cancer cell types. Cutoffs were chosen to allow for false negatives, which result from variable editing efficiencies in CRISPR screens (Fig. 1c).

To define PSODs, we first calculated the median adjusted *p* value of depletion of each gene in the eight PEL screens performed using the Brunello library. For BCBL-1, data from the clone were chosen. Genes that had a median adjusted *p* value < 0.05 were classified as PEL gene dependencies. Removing housekeeping genes from these PEL Gene Dependencies identified 210 PSODs.

### Principal component analysis

We performed PCA to investigate whether EBV(+) and EBV(−) PEL cell lines are distinguished by their genetic requirements. For these analyses, only screens that were done using the Brunello library were considered to avoid clustering based on the CRISPR library used. As input files, we used normalized read counts for aligned sgRNA reads (rlog) using the DESeq2 package^69^.

PCA were performed using the FactoMineR package^70^. Using data from all eight PEL screens, we tested sgRNAs from: (1) genes that were significantly depleted in at least one PEL cell line (FDR-adj. *p* < 0.05); or (2) PSODs. Other principal components up to PC5 were considered. None of these allowed for a separation of EBV(+) and EBV(−) cell lines. Results for PC1 and PC2 from setting (1) are shown in Fig. 1f.

### Growth curves

Clonal Cas9-expressing B cells were seeded at 2 × 10^5^ cells/mL in 500 µL in 24-well plates or 1 mL in a 12-well plates and transduced with sgRNA lentiviruses at an MOI of ~3. After 24 h, puromycin (1.2 µg/mL) was added to eliminate untransduced cells. At 3 days post transduction, absolute live cell counts were determined by fluorescence-activated cell sorting (FACS) analysis relative to a known number of spiked-in SPHERO AccuCount 5.0–5.9 µm Particles (Spherotech, Lake Forest, IL). Cells were counted every 2–3 days and cell numbers were re-adjusted to 2 × 10^5^ cells/mL at each passage, unless live cell numbers dropped below 2 × 10^5^ cells/mL, when cultures were left undisturbed. Puromycin treatment was maintained until antibiotic selection in the untransduced control cells was complete. Live cell counts for each sgRNA sample were normalized to live cell counts obtained for sgAAVS1 control transduced samples, which were set to 1. To plot viability curves, dilution factors were factored in at each passage to report cumulative live cell numbers relative to sgAAVS1. To facilitate statistical comparisons between replicate experiments, cumulative live cell numbers relative to the sgAAVS1 control at the end point of the growth curve analyses were plotted and compared. End points were set when no live cells remained, when cultures reached their minimum live cell numbers, or at 2–3 weeks into the experiment if no or only modest reductions in proliferation or viability were observed. Growth curves for different sgRNAs were done in parallel over several experiments. For this reason sgAAVS1 and sgPSMD1 data in these panels are not entirely independent. However, each experiment included these controls and data come from independent replicates in each case.

### shRNA knockdown of MCL1

Empty pGIPZ vectors, or pGIPZ expressing a scrambled shRNA control (Catalog # RHS4346) or 3′ untranslated region-directed MCL1-specific shRNAs (clone IDs: V2LHS_72724, “sh24”; and V2LHS_72721, “sh21”) were packaged using pMD2.G and psPAX2 as above. The medium was replaced 6–24 h after transfection and, 72 h after transfection, filtered virus supernatant was concentrated ~20× by ultracentrifugation (Beckman SW28 rotor, 25 000 rpm, 1 h, 4 °C). Resulting virus preparations were titrated on BJAB cells, using flow cytometry of green fluorescent protein (GFP)-positive cells, on day 2 or 3 after infection. BJAB do not respond to partial MCL1 knockdown (not shown) and are therefore suitable for accurate titration of these vectors. PEL cell lines were infected at equal MOIs, at a final concentration of 200 000 cells/mL. The actual MOIs used depend on each cell line, but were estimated to range from 1 to 5, based on the percentages of GFP-positive cells observed following infection (~60–100%). Twenty-four hours after infection, cells were collected by centrifugation and resuspended in fresh medium containing 1.5 µg/mL puromycin, a concentration that typically killed control cells within 24 h. On day 3 after transduction, a portion of the cells was harvested for western. The remaining cells were diluted by a factor of 2 and cultured for one additional day. On day 4 after transduction, FACS was used to determine absolute live cell counts as outlined above for growth curve analyses. Live cell counts were normalized to live cell counts obtained for GIPZ control transduced samples, which were set to 1.

### Western blotting

At designated time points following sgRNA lentivirus transduction, cells were collected and washed with PBS. Cells were lysed for 20 min with ice-cold RIPA buffer containing 1× protease inhibitor cocktail III (Calbiochem, EMD Millipore, Darmstadt, Germany) and 1× PhosSTOP phosphatase inhibitor cocktail (Roche, Mannheim, Germany) in 0.5 mL tubes and subjected to seven cycles of sonication (30 s on and 30 s off) in a 4 °C water bath using the Bioruptor Sonication System (Diagenode, Denville, NJ) at the high-intensity setting. Lysates were cleared by centrifugation at 14 000× *g* for 10 min at 4 °C. Protein concentrations were determined using the BCA Protein Assay Kit (Thermo Fisher Scientific). Equivalent amounts of protein (10–30 µg) were resolved in Bolt 4–12% Bis-Tris gradient gels (Thermo Fisher Scientific) and transferred to 0.22 µm nitrocellulose membranes. Specific proteins were detected following overnight incubation at 4 °C, using primary antibodies listed in Supplementary Data 15. Primary antibodies were detected with IRDye 800 CW-conjugated goat anti-rabbit or anti-mouse IgG (LI-COR Biosciences, Lincoln, NE) and imaged with the Odyssey Fc Dual-Mode Imaging System (LI-COR). MDM2 western blots were visualized with SuperSignal West Femto Maximum Sensitivity Substrate (Thermo Fisher Scientific) using horseradish peroxidase-conjugated anti-rabbit or anti-mouse IgG antibodies (Cell Signaling Technology, Danvers, MA). Uncropped western blots are found in Supplementary Figs. 9, 10.

### Determination of IC_50_ values for palbociclib and S63845

B cell lines and MEG-01 cells were seeded in eight wells of a 96-well plate at 5.6 × 10^4^ cells/mL in 90 µL volumes of complete media with 10–20% FBS (5000 cells total, Day 0). Serially diluted palbociclib (Sigma-Aldrich) or S63845 (ApexBio) were added in 10 µL volumes to seven of the wells (threefold serial dilutions from 20 to 0.027 µM final concentrations). For the negative control wells, 0.1% dimethyl sulfoxide (for S63845) or water (for palbociclib) in complete media was added. After 3 days, 20 µL of cells were harvested into a white half-area 96-well plate and lysed with 20 µL CellTiter-Glo 2.0 Reagent (Promega) for 2 min on a plate vortexer. Luminescence was read using the CellTiter-Glo program of the GloMax-Multi Detection System (Promega). IC_50_ values were calculated using the three-parameter dose response inhibitor fit in GraphPad Prism.

### Cell cycle analysis

In all, 2–5×10^5^ PEL cells were harvested and washed with ice-cold PBS. Cells were fixed and permeabilized with 500 µL ice-cold 70% ethanol in PBS for at least 1 h at −20 °C. After fixation, cells were washed with PBS and stained with 300 µL propidium iodide/RNase staining buffer (BD Pharmigen) at 4 °C in the dark for at least 15 min. Stained cells were immediately analyzed for propidium iodide fluorescence using BD FACSCanto II. Cell cycle analysis was performed using the Cell Cycle platform in FlowJo v10. Model fittings were done with either the Watson Pragmatic algorithm or Dean-Jett-Fox algorithm with unconstrained or constrained settings (G1 × 2), minimizing the root mean square error.

### Immunohistochemical staining on PEL tumor sections

After approval by the Institutional Review Board of Northwestern University, pathology records were searched for cases with the diagnosis of PEL between January 2010 and April 2016. A total of nine cases from four patients were identified (Supplementary Data 16). All of the samples were cytology cell blocks from pleural effusion or peritoneal fluid. Medical records of the patients, as well their available pathology slides were reviewed by a pathologist (A.B.). LANA immunohistochemical (IHC) stain (mouse monoclonal antibody; Cell Marque; 265M-18) and EBER in situ hybridization (EBER1 DNP probe; Ventana; 760–1209) were previously performed as part of patient’s routine diagnostic work up at the time of first diagnosis. MCL1 IHC stain was performed as part of this project at Northwestern Pathology Core facility. Briefly, 5 µm sections of the formalin-fixed paraffin-embedded cytology cell blocks were used for these stains. IHC was performed using a monoclonal antibody against MCL-1 (Cell Signaling 39224) at a dilution of 1:200. Tonsillar specimens were utilized as controls.

### Data availability

The deep-sequencing data for the CRISPR screens are available in SRA SRP081136.

## Electronic supplementary material

Supplementary Information

Description of Additional Supplementary Files

Supplementary Data 1

Supplementary Data 2

Supplementary Data 3

Supplementary Data 4

Supplementary Data 5

Supplementary Data 6

Supplementary Data 7

Supplementary Data 8

Supplementary Data 9

Supplementary Data10

Supplementary Data 11

Supplementary Data 12

Supplementary Data 13

Supplementary Data 14

Supplementary Data 15

Supplementary Data 16

## References

[1] Nador RG (1996). Primary effusion lymphoma: a distinct clinicopathologic entity associated with the Kaposi’s sarcoma-associated herpes virus. Blood.

[2] Cesarman E, Chang Y, Moore PS, Said JW, Knowles DM (1995). Kaposi’s sarcoma-associated herpesvirus-like DNA sequences in AIDS-related body-cavity-based lymphomas. N. Engl. J. Med..

[3] Chang Y (1994). Identification of herpesvirus-like DNA sequences in AIDS-associated Kaposi’s sarcoma. Science.

[4] Soulier J (1995). Kaposi’s sarcoma-associated herpesvirus-like DNA sequences in multicentric Castleman’s disease. Blood.

[5] Cesarman E (2014). Gammaherpesviruses and lymphoproliferative disorders. Annu. Rev. Pathol..

[6] Okada S, Goto H, Yotsumoto M (2014). Current status of treatment for primary effusion lymphoma. Intractable Rare Dis. Res..

[7] Boulanger E (2005). Prognostic factors and outcome of human herpesvirus 8-associated primary effusion lymphoma in patients with AIDS. J. Clin. Oncol..

[8] Katano H, Sato Y, Sata T (2001). Expression of p53 and human herpesvirus-8 (HHV-8)-encoded latency-associated nuclear antigen with inhibition of apoptosis in HHV-8-associated malignancies. Cancer.

[9] Petre CE, Sin SH, Dittmer DP (2007). Functional p53 signaling in Kaposi’s sarcoma-associated herpesvirus lymphomas: implications for therapy. J. Virol..

[10] Cesarman E (1995). In vitro establishment and characterization of two acquired immunodeficiency syndrome-related lymphoma cell lines (BC-1 and BC-2) containing Kaposi’s sarcoma-associated herpesvirus-like (KSHV) DNA sequences. Blood.

[11] Trivedi P (2004). Infection of HHV-8+ primary effusion lymphoma cells with a recombinant Epstein-Barr virus leads to restricted EBV latency, altered phenotype, and increased tumorigenicity without affecting TCL1 expression. Blood.

[12] McHugh D (2017). Persistent KSHV infection increases EBV-associated tumor formation in vivo via enhanced EBV lytic gene expression. Cell Host Microbe.

[13] Godfrey A, Anderson J, Papanastasiou A, Takeuchi Y, Boshoff C (2005). Inhibiting primary effusion lymphoma by lentiviral vectors encoding short hairpin RNA. Blood.

[14] Guasparri I, Keller SA, Cesarman E (2004). KSHV vFLIP is essential for the survival of infected lymphoma cells. J. Exp. Med..

[15] Wies, E. et al. The viral interferon-regulatory factor-3 is required for the survival of KSHV-infected primary effusion lymphoma cells. *Blood* **111**, 320–327 (2008). 10.1182/blood-2007-05-092288 17890449

[16] Chen W, Hilton IB, Staudt MR, Burd CE, Dittmer DP (2010). Distinct p53, p53:LANA, and LANA complexes in Kaposi’s sarcoma-associated herpesvirus lymphomas. J. Virol..

[17] Sarek G (2007). Reactivation of the p53 pathway as a treatment modality for KSHV-induced lymphomas. J. Clin. Invest..

[18] Santag S (2013). Recruitment of the tumour suppressor protein p73 by Kaposi’s Sarcoma Herpesvirus latent nuclear antigen contributes to the survival of primary effusion lymphoma cells. Oncogene.

[19] Rivas C, Thlick AE, Parravicini C, Moore PS, Chang Y (2001). Kaposi’s sarcoma-associated herpesvirus LANA2 is a B-cell-specific latent viral protein that inhibits p53. J. Virol..

[20] Keller SA, Schattner EJ, Cesarman E (2000). Inhibition of NF-kappa B induces apoptosis of KSHV-infected primary effusion lymphoma cells. Blood.

[21] Field N (2003). KSHV vFLIP binds to IKK-gamma to activate IKK. J. Cell Sci..

[22] Thome M (1997). Viral FLICE-inhibitory proteins (FLIPs) prevent apoptosis induced by death receptors. Nature.

[23] Chaudhary PM, Jasmin A, Eby MT, Hood L (1999). Modulation of the NF-kappa B pathway by virally encoded death effector domains-containing proteins. Oncogene.

[24] Tolani B, Matta H, Gopalakrishnan R, Punj V, Chaudhary PM (2014). NEMO is essential for Kaposi’s sarcoma-associated herpesvirus-encoded vFLIP K13-induced gene expression and protection against death receptor-induced cell death, and its N-terminal 251 residues are sufficient for this process. J. Virol..

[25] Cousins E, Nicholas J (2014). Molecular biology of human herpesvirus 8: novel functions and virus-host interactions implicated in viral pathogenesis and replication. Recent Results Cancer Res..

[26] Sin SH (2007). Rapamycin is efficacious against primary effusion lymphoma (PEL) cell lines in vivo by inhibiting autocrine signaling. Blood.

[27] Uddin S (2005). Inhibition of phosphatidylinositol 3’-kinase/AKT signaling promotes apoptosis of primary effusion lymphoma cells. Clin. Cancer Res..

[28] Li M (1997). Kaposi’s sarcoma-associated herpesvirus encodes a functional cyclin. J. Virol..

[29] Swanton C (1997). Herpes viral cyclin/Cdk6 complexes evade inhibition by CDK inhibitor proteins. Nature.

[30] Fan W (2005). Distinct subsets of primary effusion lymphoma can be identified based on their cellular gene expression profile and viral association. J. Virol..

[31] Jenner RG (2003). Kaposi’s sarcoma-associated herpesvirus-infected primary effusion lymphoma has a plasma cell gene expression profile. Proc. Natl Acad. Sci. USA.

[32] Klein U (2003). Gene expression profile analysis of AIDS-related primary effusion lymphoma (PEL) suggests a plasmablastic derivation and identifies PEL-specific transcripts. Blood.

[33] Shaffer AL (2008). IRF4 addiction in multiple myeloma. Nature.

[34] Gopalakrishnan R, Matta H, Tolani B, Triche T, Chaudhary PM (2016). Immunomodulatory drugs target IKZF1-IRF4-MYC axis in primary effusion lymphoma in a cereblon-dependent manner and display synergistic cytotoxicity with BRD4 inhibitors. Oncogene.

[35] Doudna JA, Charpentier E (2014). Genome editing. The new frontier of genome engineering with CRISPR-Cas9. Science.

[36] Shalem O, Sanjana NE, Zhang F (2015). High-throughput functional genomics using CRISPR-Cas9. Nat. Rev. Genet..

[37] Doench JG (2016). Optimized sgRNA design to maximize activity and minimize off-target effects of CRISPR-Cas9. Nat. Biotechnol..

[38] Sanjana NE, Shalem O, Zhang F (2014). Improved vectors and genome-wide libraries for CRISPR screening. Nat. Methods.

[39] Li W (2015). Quality control, modeling, and visualization of CRISPR screens with MAGeCK-VISPR. Genome Biol..

[40] Aguirre AJ (2016). Genomic copy number dictates a gene-independent cell response to CRISPR/Cas9 targeting. Cancer Discov..

[41] Ma Y (2017). CRISPR/Cas9 screens reveal Epstein-Barr virus-transformed B cell host dependency factors. Cell Host Microbe.

[42] Wang T (2015). Identification and characterization of essential genes in the human genome. Science.

[43] Wang T (2017). Gene essentiality profiling reveals gene networks and synthetic lethal interactions with oncogenic Ras. Cell.

[44] Subramanian A (2005). Gene set enrichment analysis: a knowledge-based approach for interpreting genome-wide expression profiles. Proc. Natl Acad. Sci. USA.

[45] Cojohari O (2015). BH3 profiling reveals selectivity by herpesviruses for specific Bcl-2 proteins to mediate survival of latently infected cells. J. Virol..

[46] Nayar U (2013). Targeting the Hsp90-associated viral oncoproteome in gammaherpesvirus-associated malignancies. Blood.

[47] Lagunoff M (2016). Activation of cellular metabolism during latent Kaposi’s sarcoma herpesvirus infection. Curr. Opin. Virol..

[48] Hart T (2015). High-resolution CRISPR screens reveal fitness genes and genotype-specific cancer liabilities. Cell.

[49] Wang T, Wei JJ, Sabatini DM, Lander ES (2014). Genetic screens in human cells using the CRISPR-Cas9 system. Science.

[50] Chesi M (1996). Dysregulation of cyclin D1 by translocation into an IgH gamma switch region in two multiple myeloma cell lines. Blood.

[51] O’Neill KL, Huang K, Zhang J, Chen Y, Luo X (2016). Inactivation of prosurvival Bcl-2 proteins activates Bax/Bak through the outer mitochondrial membrane. Genes Dev..

[52] Beroukhim R (2010). The landscape of somatic copy-number alteration across human cancers. Nature.

[53] Kotschy A (2016). The MCL1 inhibitor S63845 is tolerable and effective in diverse cancer models. Nature.

[54] Krajewski S (1995). Immunohistochemical analysis of Mcl-1 protein in human tissues. Differential regulation of Mcl-1 and Bcl-2 protein production suggests a unique role for Mcl-1 in control of programmed cell death in vivo. Am. J. Pathol..

[55] Keller SA (2006). NF-kappaB is essential for the progression of KSHV- and EBV-infected lymphomas in vivo. Blood.

[56] Rauert-Wunderlich H (2013). The IKK inhibitor Bay 11-7082 induces cell death independent from inhibition of activation of NFkappaB transcription factors. PLoS ONE.

[57] White DE, Burchill SA (2008). BAY 11-7082 induces cell death through NF-kappaB-independent mechanisms in the Ewing’s sarcoma family of tumours. Cancer Lett..

[58] Lee J, Rhee MH, Kim E, Cho JY (2012). BAY 11-7082 is a broad-spectrum inhibitor with anti-inflammatory activity against multiple targets. Mediat. Inflamm..

[59] Grumont RJ, Gerondakis S (2000). Rel induces interferon regulatory factor 4 (IRF-4) expression in lymphocytes: modulation of interferon-regulated gene expression by rel/nuclear factor kappaB. J. Exp. Med..

[60] Chen C, Edelstein LC, Gelinas C (2000). The Rel/NF-kappaB family directly activates expression of the apoptosis inhibitor Bcl-x(L). Mol. Cell. Biol..

[61] Lee JS (2009). FLIP-mediated autophagy regulation in cell death control. Nat. Cell Biol..

[62] Sarek G, Ojala PM (2007). p53 reactivation kills KSHV lymphomas efficiently in vitro and in vivo: new hope for treating aggressive viral lymphomas. Cell Cycle.

[63] Goncalves PH, Uldrick TS, Yarchoan R (2017). HIV-associated Kaposi sarcoma and related diseases. AIDS.

[64] Patil, A., Manzano, M. & Gottwein, E. CK1α and IRF4 are essential and independent effectors of immunomodulatory drugs in primary effusion lymphoma. *Blood*10.1182/blood-2018-01-828418 (2018). 10.1182/blood-2018-01-828418 PMC6085990 29954751

[65] Shalem O (2014). Genome-scale CRISPR-Cas9 knockout screening in human cells. Science.

[66] Hsu PD (2013). DNA targeting specificity of RNA-guided Cas9 nucleases. Nat. Biotechnol..

[67] Chen S (2015). Genome-wide CRISPR screen in a mouse model of tumor growth and metastasis. Cell.

[68] Reddy A (2017). Genetic and functional drivers of diffuse large B cell lymphoma. Cell.

[69] Love MI, Huber W, Anders S (2014). Moderated estimation of fold change and dispersion for RNA-seq data with DESeq2. Genome Biol..

[70] Le S, Josse J, Husson F (2008). FactoMineR: an R package for multivariate analysis. J. Stat. Softw..

