# Supplementary material for: Gene essentiality landscape and druggable oncogenic dependencies in herpesviral primary effusion lymphoma
Source: Nat Commun. 2018 Aug 15;9:3263. doi: 10.1038/s41467-018-05506-9 (PMC6093911; doi:10.1038/s41467-018-05506-9)
Supplement: Supplementary file 1 — Supplementary Information [file 41467_2018_5506_MOESM1_ESM.pdf]

Supplementary Information

Gene Essentiality Landscape and Druggable Oncogenic Dependencies in Herpesviral Primary Effusion Lymphoma

Mark Manzano[1], Ajinkya Patil[1], Alexander Waldrop[2], Sandeep S. Dave[2], Amir Behdad[3], and Eva Gottwein[1]

[1]Department of Microbiology-Immunology and [3]Department of Pathology, Feinberg School of Medicine, Northwestern University, Chicago, Illinois, 60611 USA.

[2]Duke Cancer Institute and Center for Genomic and Computational Biology, Duke University, Durham, NC, 27708 USA

Corresponding author: **Eva Gottwein (e-gottwein@northwestern.edu)**

Includes:

Supplementary Figure 1. Significant sgRNA depletion in CRISPR screens.

Supplementary Figure 2. Preferentially required genes in either EBV(+) or EBV(-) PEL cell lines.

Supplementary Figure 3. Comparison of sgRNA depletion in Brunello screens of BCBL-1 Cas9 cell pool and a cell clone derived from this pool.

Supplementary Figure 4. Details of growth curve analyses for validation studies.

Supplementary Figure 5. Validation experiments for NF-κB signaling components.

Supplementary Figure 6. Cell cycle analysis of propidium iodide-stained BC-3 cells treated for 24 h with 1.5 μM palbociclib (IC50).

Supplementary Figure 7. Differential dependence of BC-3 and BCBL-1 cells on BCL2L1 (Bcl-xL).

Supplementary Figure 8. shRNA-mediated knockdown of MCL1 in a wider panel of PEL cell lines.

Supplementary Figure 9. Uncropped Western blots for Main Figures.

Supplementary Figure 10. Uncropped Western blots for Supplementary Figures.

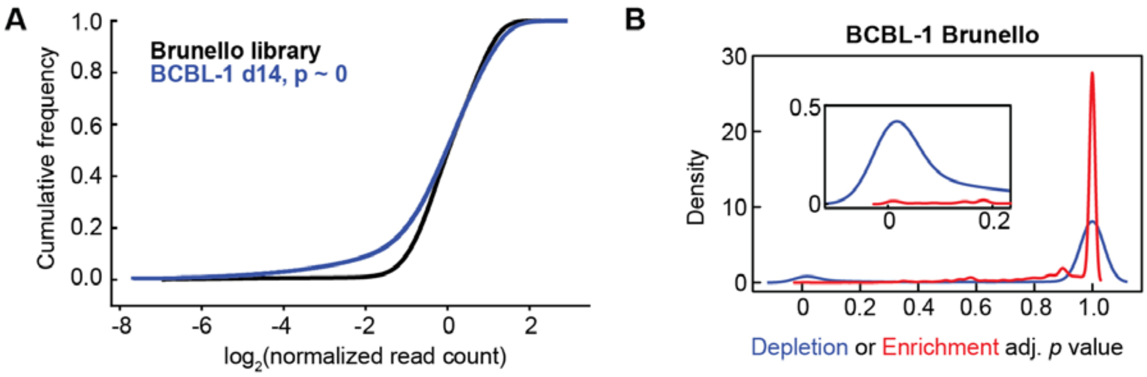

Supplementary Figure 1. Significant sgRNA depletion in CRISPR screens. Representative data from BCBL-1 Cas9 pool screened using Brunello library. (A) Cumulative frequency distribution plot of normalized sgRNA counts. *p* value was determined by two-sample Kolmogorov-Smirnov test. (B) Density plots show the distribution of adj. *p* value for depletion (red) or enrichment (blue) at the gene level. The area containing high-confidence hits (low adj. *p* values) is magnified in inset.

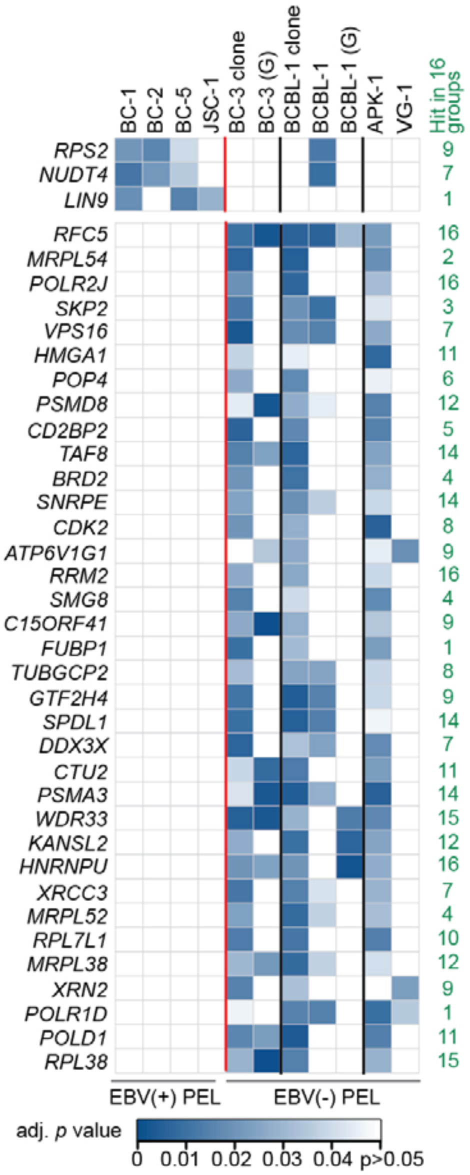

Supplementary Figure 2. Preferentially required genes in either EBV(+) or EBV(-) PEL cell lines. Heatmap of adj. p values of sgRNA depletion for candidates for genes that are preferentially essential in EBV(+) or EBV(-) PEL cell lines. Genes that were significantly depleted (adj. $p < 0.05$) in ≥3/4 EBV+ PEL cells but not in EBV(-) PEL cells were considered preferentially essential in the former group (top). Genes that were significantly depleted (adj. $p < 0.05$) in ≥3/4 EBV(-) PEL cells but not in EBV(+) PEL cells were considered preferentially essential in EBV(-) PELs (bottom). For BC-3 and BCBL-1 cell lines, only BC-3 (Brunello) and BCBL-1 clone (Brunello) screens were used for these comparisons. Indicated on the right are the numbers of cancer types where the gene was found to be potentially essential. The Brunello library was used for most of the screens except where indicated: G, GeCKO v2.

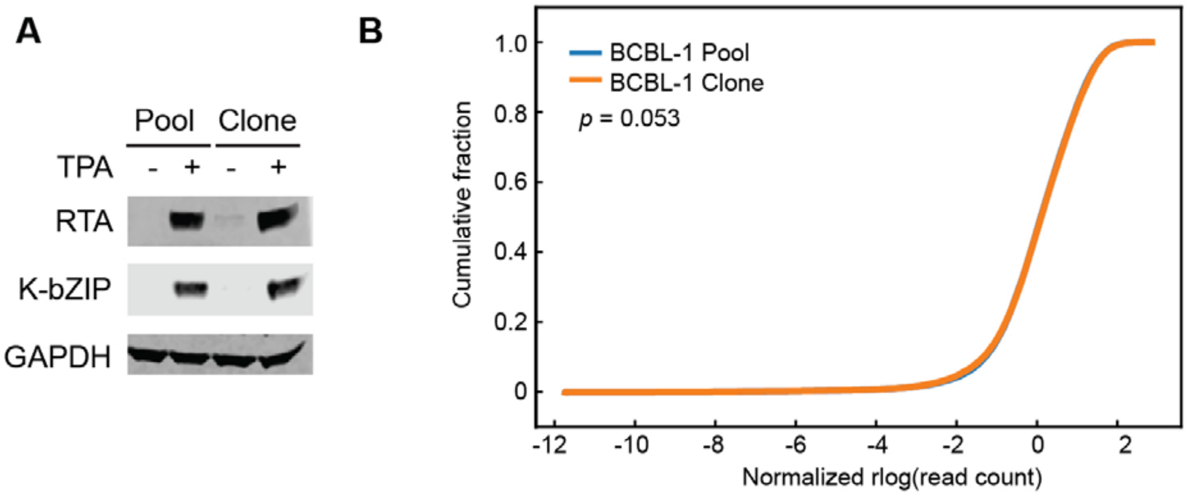

Supplementary Figure 3. Comparison of sgRNA depletion in Brunello screens of BCBL-1 Cas9 cell pool and a cell clone derived from this pool. (A) Western blot for viral lytic proteins upon TPA-induced lytic reactivation. (B) Cumulative frequency distribution plot of normalized sgRNA counts from d14 samples from BCBL-1 Cas9 cell pool (blue line) or clone (orange). *p* value was determined by two-sample Kolmogorov-Smirnov test.

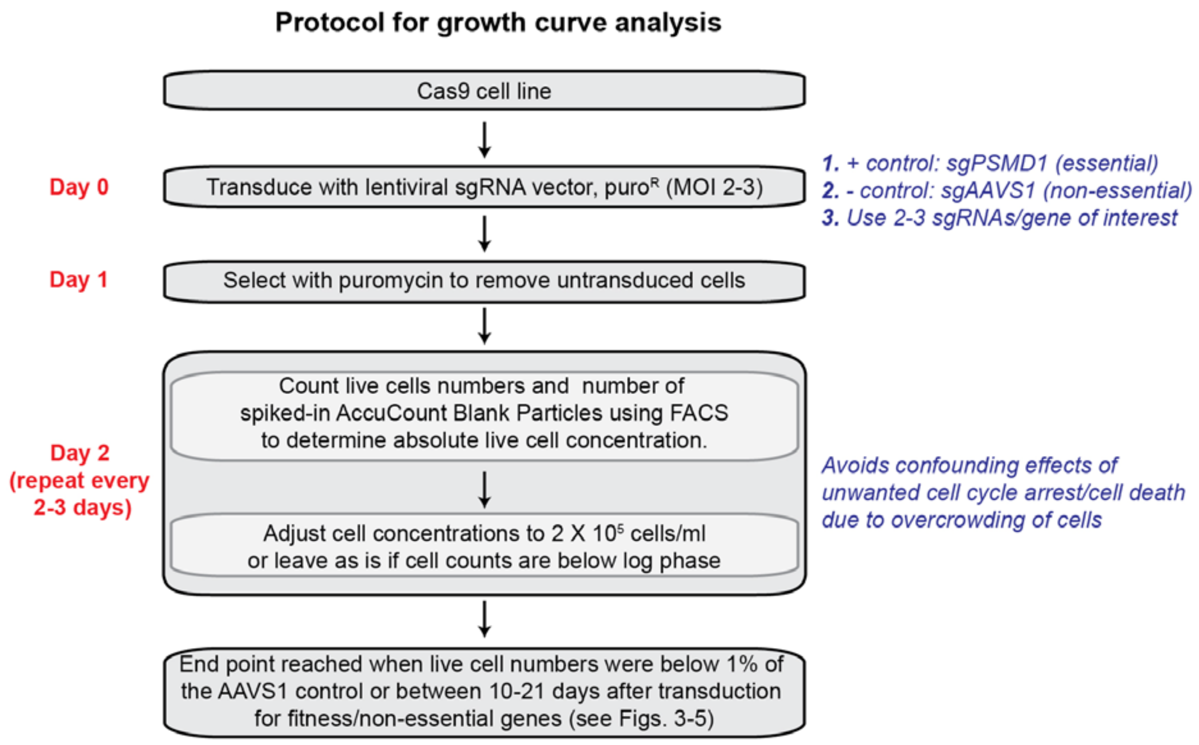

Supplementary Figure 4. Details of growth curve analyses for validation studies. Validation experiments were performed in clonal Cas9 expressing B cell lines with optimal gene editing. We used sgAAVS1, which targets the non-coding AAVS1 locus, as a negative control. sgPSMD1, which targets an essential proteasomal subunit, served as a positive control. Live cell numbers were plotted relative to the sgAAVS1 control over time. Editing of different genes resulted in a loss of live cells at different time points into the experiment. We therefore report the cumulative live cell counts relative to the sgAAVS1 control on the final day of the experiment over independent experiments to facilitate data presentation and statistical analysis.

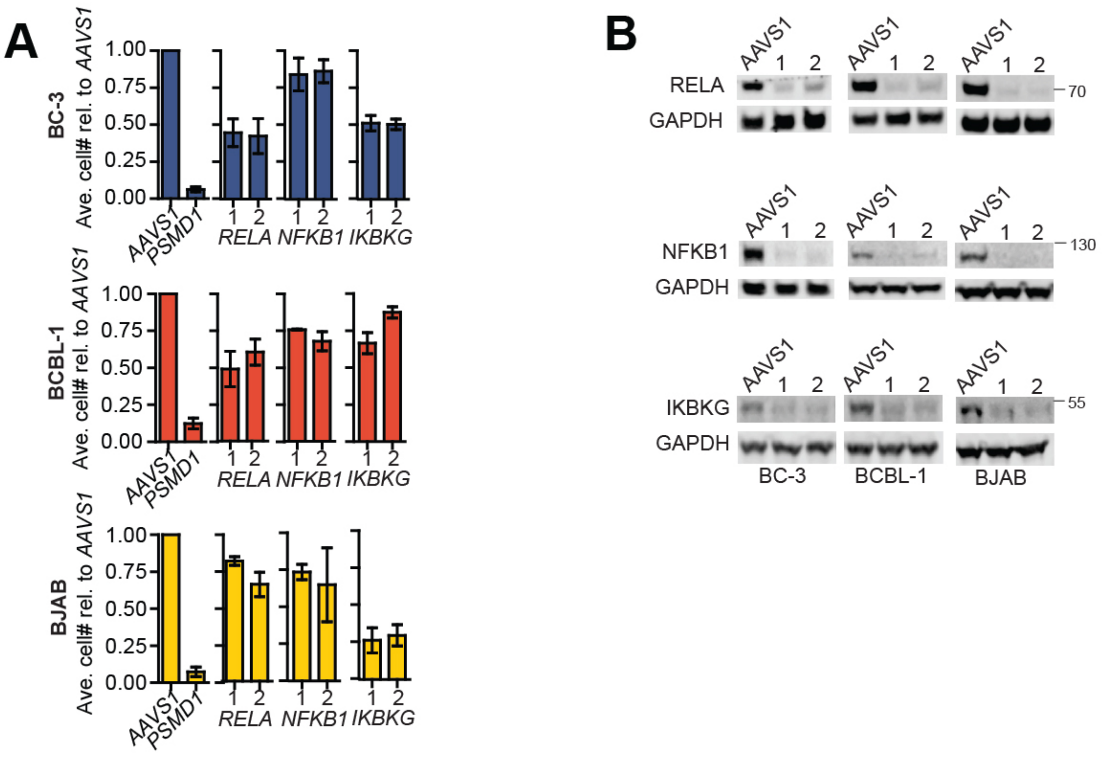

Supplementary Figure 5. Validation experiments for NF-κB signaling components. (A) End point analysis of several growth curve assays following the individual knockout of RELA (p65), NFKB1 (p50), or IKBKG (Nemo) in Cas9-expressing BC-3, BCBL-1 or BJAB cell clones. (B) Representative Western blots of cells in (A). AAVS1, control sgRNA targeting the non-coding AAVS1 locus; PSMD1, control sgRNA targeting the housekeeping proteasome subunit. Error bars represent SEM, n≥3.

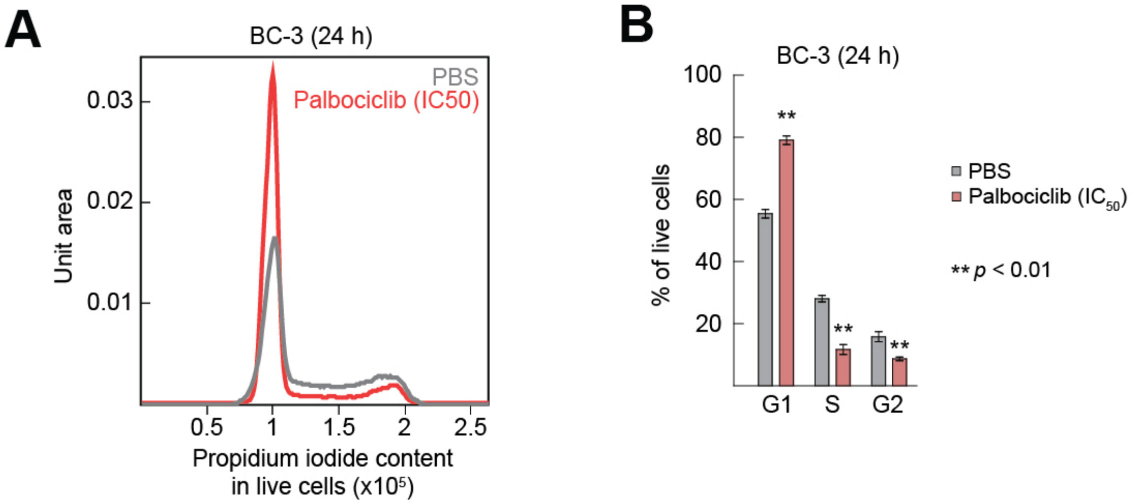

Supplementary Figure 6. Cell cycle analysis of propidium iodide-stained BC-3 cells treated for 24 h with 1.5 μM palbociclib (IC50). (A) Representative histogram of DNA content from propidium iodide-staining. (B) Distribution of cell cycle phase populations. *p* values were calculated by Student's t test and compared to PBS-treated cells in the same phase. n=4. All error bars, SEM.

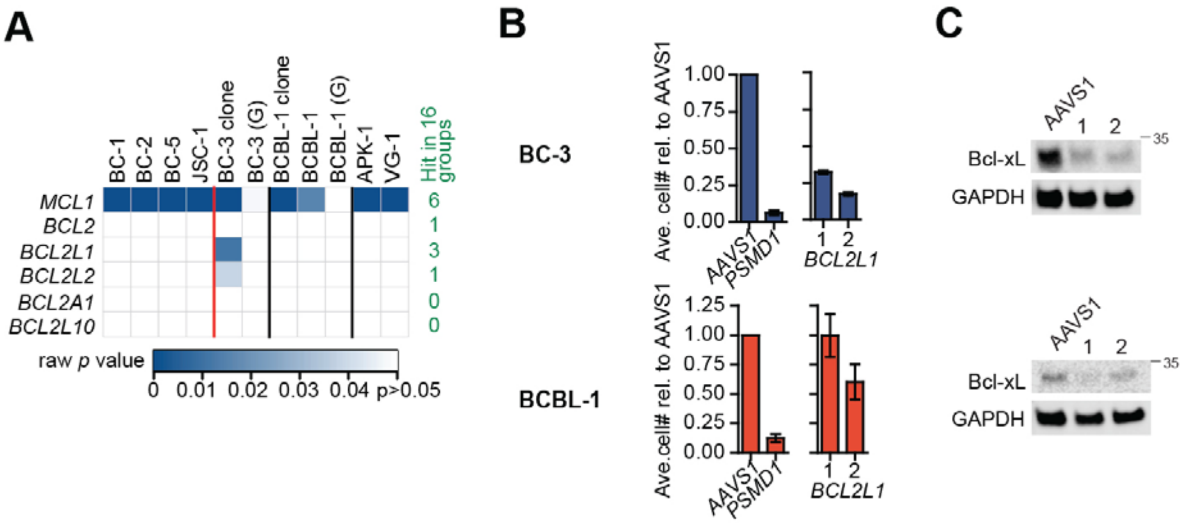

Supplementary Figure 7. Differential dependence of BC-3 and BCBL-1 cells on BCL2L1 (Bcl-xL). (A) Heatmap of raw *p* values of sgRNA depletion of the BCL2 family in cell lines screened. Indicated on the right are the numbers of cancer types (out of 16) where the gene was found to be a potential dependency. The Brunello library was used for most of the screens except where indicated: G, GeCKO v2. (B) End point of several growth curve analyses following BCL2L1 knockout in BC-3 or BCBL-1 Cas9 cells. (C) Representative Western blots for Bcl-xL in (B). Error bars, SEM; n≥3.

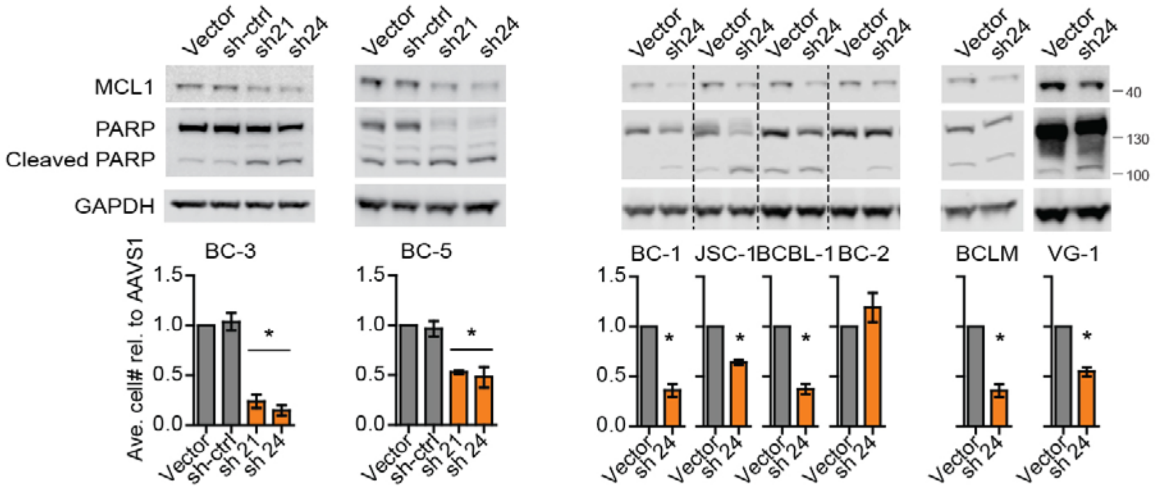

Supplementary Figure 8. shRNA-mediated knockdown of MCL1 in a wider panel of PEL cell lines. Representative Western blots for MCL1 and PARP cleavage at 3 days post-transduction of lentiviral sgRNA are shown on top; summary of live cell counts at 4 days post-transduction of lentiviral sgRNA is shown below. *$p < 0.05$ by Student's t test. All error bars, SEM.

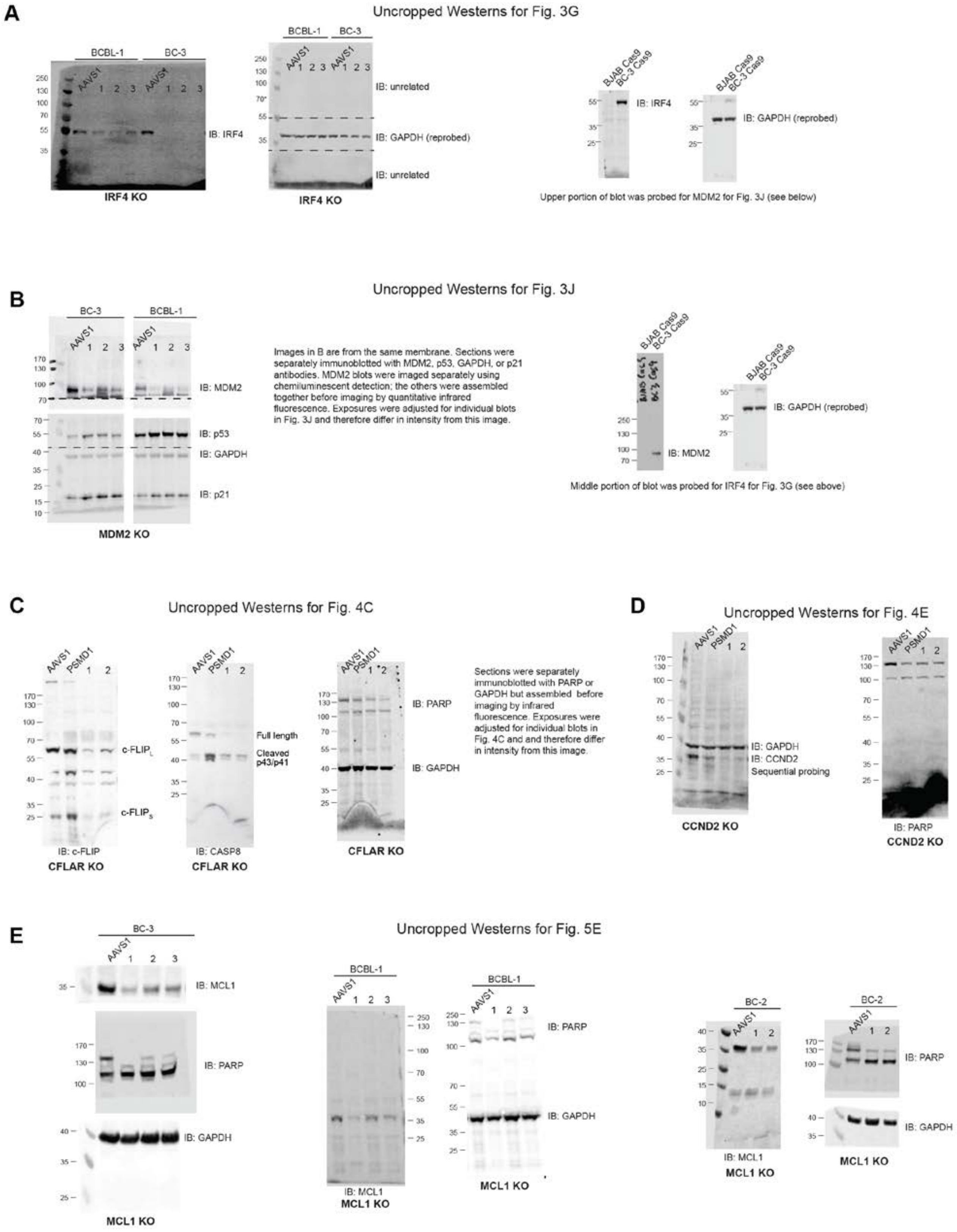

Supplementary Figure 9. Uncropped Western blots for Main Figures. (A) Fig. 3G, (B) Fig. 3J, (C) Fig. 4C, (D) Fig. 4E, (E) Fig. 5E. Molecular weight markers are shown on each blot. IB: immunoblot antibody.

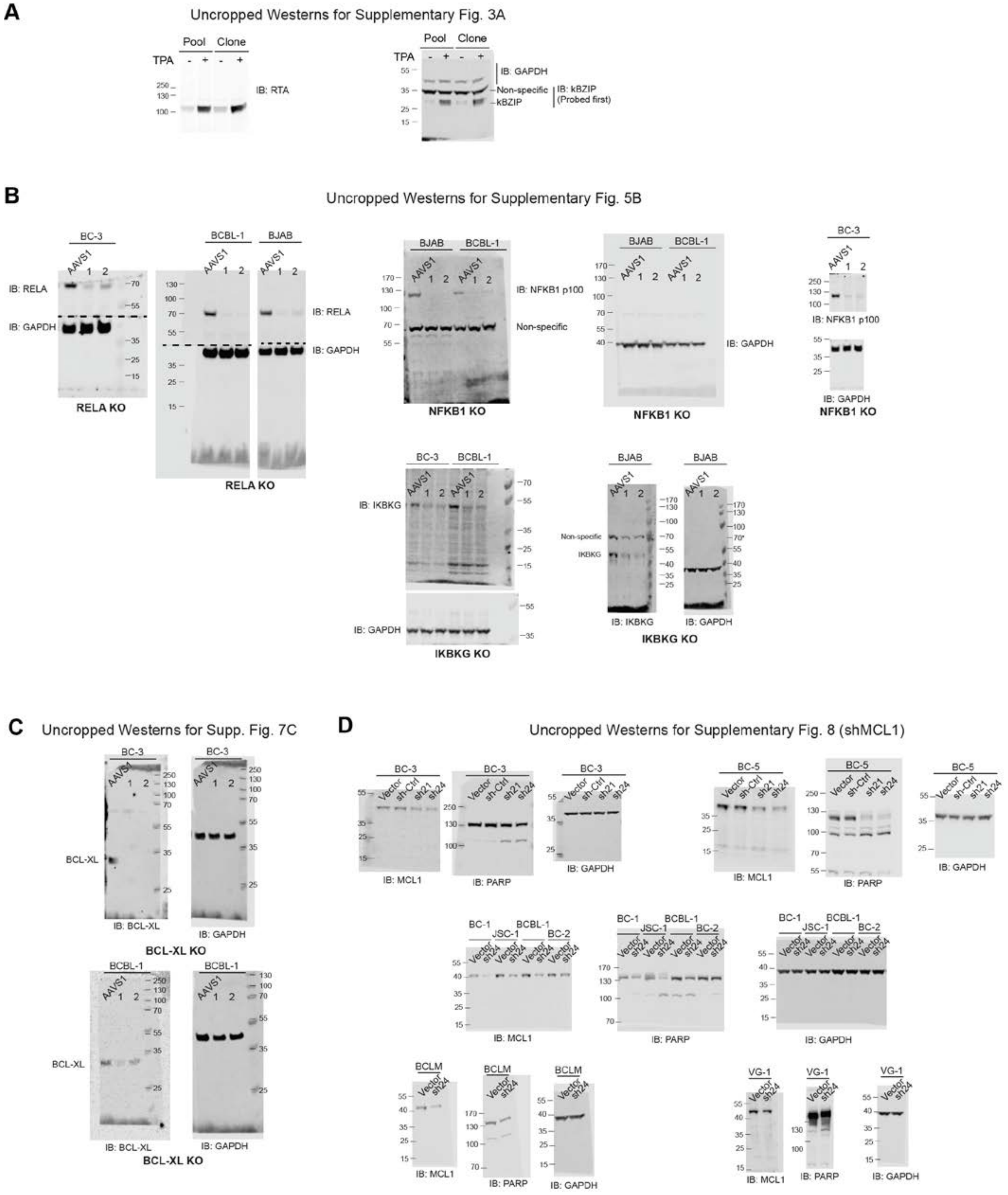

Supplementary Figure 10. Uncropped Western blots for Supplementary Figures (A) 3A, (B) 5B, (C) 7C, (D) 8. Molecular weight markers are shown on each blot. IB: immunoblot antibody.
