# Supplementary material for: Gene essentiality landscape and druggable oncogenic dependencies in herpesviral primary effusion lymphoma
Source: Nat Commun. 2018 Aug 15;9:3263. doi: 10.1038/s41467-018-05506-9 (PMC6093911; doi:10.1038/s41467-018-05506-9)
Supplement: Supplementary file 2 — Description of Additional Supplementary Files [file 41467_2018_5506_MOESM2_ESM.pdf]

Supplemental Data Legends

Supplementary Data 1. Sixty CRISPR screens performed in this study or reanalyzed from published data were used to define housekeeping genes or PEL specific oncogenic dependencies (PSODs), as outlined in Fig. 2B. Shown are cancer cell type (Group), source of the CRISPR screen data, Cell line name, the number of genes with FDR-adjusted *p* value of depletion <0.05 as calculated by MAGeCK, and the sgRNA library used in each screen.

Supplementary Data 2. Raw sgRNA read counts for the screens performed in this study. sgRNAs targeting genes that were flagged to be potentially amplified by Sliding Window Analysis have been removed from the lists and are listed in Supplementary Table 3. (A) sgRNA raw read counts for PEL cell lines screened with the Brunello library; (B) sgRNA raw read counts for KMS-12-BM; (C, D) sgRNA raw read counts for cell lines screened with the GECKO library, (E-F) sgRNA IDs and sequences found in each library.

Supplementary Data 3. Flagging of potentially amplified regions using Sliding Window Analysis. (A) Genes removed from analysis after Sliding Window Analysis. Gene = official gene symbol, max_neighborhood_score = maximum 40-gene neighborhood sliding window score that includes the gene, crispr_score = quantifies gene knockout effect, chr = chromosome, start = starting position of furthest upstream sgRNA targeting the gene, end = starting position of furthest downstream sgRNA targeting the gene, Cell_Line = cell line where gene should be removed from downstream analysis, Sanger_CN = COSMIC v71 copy number of gene in cell line. (B, C) CRISPR scores generated as part of this analysis.

Supplementary Data 4. Gene-level output files from MAGeCK for Brunello and GECKO screens. Tests for depletion or enrichment of sgRNAs for genes are shown as “neg” or “pos”, respectively. Legend: id = gene symbol, num = number of sgRNAs per gene; score = RRA lo value, p-value = raw *p* value (permutation), fdr = false discovery rate-adjusted p value, rank = rank of gene based on significance of depletion/enrichment, goodsgrna = number of sgRNAs whose ranking is below the alpha cutoff, lfc = median $\log_2$ fold change of sgRNA.

Supplementary Data 5. sgRNA-level output files from MAGeCK from Brunello screens. Legend: sgrna = sgRNA ID, gene = official gene symbol, control_count = normalized read counts of control samples, treatment_count = normalized read counts of treatment samples, control_mean = mean normalized read counts of control samples, treat_mean = mean normalized read counts of treatment samples, LFC = log2 fold change, control_var = raw variance in control samples, adj_var = adjusted variance in control samples, score = sgRNA score, p.low = *p* value (lower tail), p.high = *p* value (higher tail), p.twosided = *p* value (two-sided), FDR = false discovery rate-adjusted *p* value, high_in_treatment = if sgRNA is higher in treatment samples compared to control samples.

Supplementary Data 6. sgRNA-level output files from MAGeCK from GECKO screens. Legend: sgrna = sgRNA ID, gene = official gene symbol, control_count = normalized read counts of control samples, treatment_count = normalized read counts of treatment samples, control_mean = mean normalized read counts of control samples, treat_mean = mean normalized read counts of treatment samples, LFC = log2 fold change, control_var = raw variance in control samples, adj_var = adjusted variance in control samples, score = sgRNA score, p.low = *p* value (lower tail), p.high = *p* value (higher tail), p.twosided = *p* value (two-sided), FDR = false discovery rate-adjusted *p* value, high_in_treatment = if sgRNA is higher in treatment samples compared to control samples.

Supplementary Data 7. Gene Set Enrichment Analysis results for each PEL screen using the curated gene sets from the Reactome Pathway Database as summarized in Fig. 1E.

Supplementary Data 8. Summarized results for negative selection of all genes screen in Brunello and GECKO libraries. Adjusted p values of each gene in each cell line screened are shown. The median adjusted p values of genes and their corresponding ranks from the eight PEL cell lines screened using the Brunello library are calculated. For BCBL-1, data from the BCBL-1 clone was used for this analysis. Also included is information on in how many cancer groups (out of 16) each gene is considered potentially essential (see Supplementary Methods for details).

Supplementary Data 9. List of potential housekeeping genes. Genes shown here had a median adjusted p value of depletion of <0.25 within each cancer type in at least 10 of 16 cancer types.

Supplementary Data 10. Ranked list of genes classified as PSODs. This list includes both likely essential genes and fitness genes.

Supplementary Data 11. Pathway enrichment analyses using DAVID of PSODs.

Supplementary Data 12. Columns indicate the sgRNA sequences for all sgRNAs used in this study, as well as forward and reverse primers used for insertion into plentiGuide-puro.

Supplementary Data 13. Primers used for next generation sequencing library construction.

Supplementary Data 14. Library and read statistics.

Supplementary Data 15. List of antibodies used in this study.

Supplementary Data 16. Information for PEL patients for tumor sections in Figure 6. Four cases were identified from pathology records at the Northwestern Memorial Hospital between January 2010 and April 2016. Presence of KSHV or EBV was determined using immunohistochemical staining of LANA or in situ hybridization for EBER RNA at the time of diagnosis.
